# Probiotics, prebiotics, synbiotics and other microbiome-based innovative therapeutics to mitigate obesity and enhance longevity via the gut-brain axis

**DOI:** 10.20517/mrr.2024.05

**Published:** 2024-05-17

**Authors:** Jacqueline L. Boyajian, Paromita Islam, Ahmed Abosalha, Sabrina Schaly, Rahul Thareja, Amal Kassab, Karan Arora, Madison Santos, Cedrique Shum-Tim, Satya Prakash

**Affiliations:** ^1^Biomedical Technology and Cell Therapy Research Laboratory, Department of Biomedical Engineering, Faculty of Medicine and Health Sciences, McGill University, Montreal H3A 2B4, Quebec, Canada.; ^2^Pharmaceutical Technology Department, Faculty of Pharmacy, Tanta University, Tanta 31111, Egypt.

**Keywords:** Microbiome, obesity, aging, gut-brain axis, prebiotics, probiotics, postbiotics, therapeutics

## Abstract

The global prevalence of obesity currently exceeds 1 billion people and is accompanied by an increase in the aging population. Obesity and aging share many hallmarks and are leading risk factors for cardiometabolic disease and premature death. Current anti-obesity and pro-longevity pharmacotherapies are limited by side effects, warranting the development of novel therapies. The gut microbiota plays a major role in human health and disease, with a dysbiotic composition evident in obese and aged individuals. The bidirectional communication system between the gut and the central nervous system, known as the gut-brain axis, may link obesity to unhealthy aging. Modulating the gut with microbiome-targeted therapies, such as biotics, is a novel strategy to treat and/or manage obesity and promote longevity. Biotics represent material derived from living or once-living organisms, many of which have therapeutic effects. Pre-, pro-, syn- and post-biotics may beneficially modulate gut microbial composition and function to improve obesity and the aging process. However, the investigation of biotics as next-generation therapeutics has only just begun. Further research is needed to identify therapeutic biotics and understand their mechanisms of action. Investigating the function of the gut-brain axis in obesity and aging may lead to novel therapeutic strategies for obese, aged and comorbid (e.g., sarcopenic obese) patient populations. This review discusses the interrelationship between obesity and aging, with a particular emphasis on the gut microbiome, and presents biotics as novel therapeutic agents for obesity, aging and related disease states.

## INTRODUCTION

**Figure 1:**
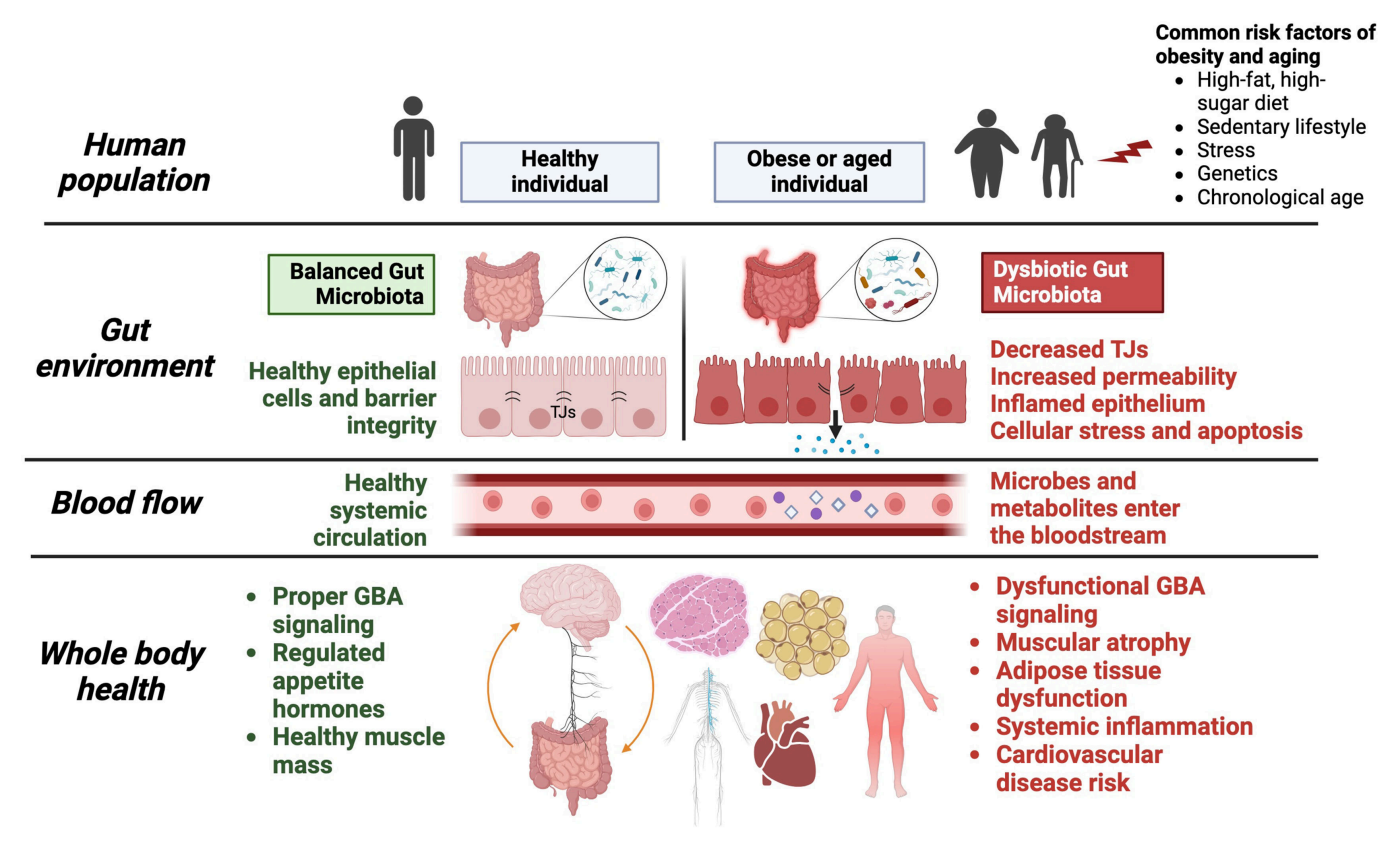
A comparison of gut microbiota, intestinal epithelium, and systemic health in healthy versus obese or aged individuals. A balanced gut microbiome maintains host health homeostasis, while a dysbiotic gut leads to an impaired intestinal barrier and harmful systemic consequences, as evident in obesity and aging. The downstream effects of gut dysbiosis, such as inflammation, adipose tissue dysfunction, and muscular atrophy, promote obese and aging phenotypes. TJs: Tight junctions; GBA: gut-brain axis.

Obesity is a complex, multifactorial and chronic disease that currently affects over 1 billion adults, adolescents, and children worldwide. As the prevalence of overweight or obese adults continues to rise, there is a parallel increase observed within the aging population^[1]^. Aging is the leading risk factor for major debilitating and life-threatening diseases. Obesity and aging share many biological hallmarks, including systemic inflammation and oxidative stress, increased adiposity, skeletal muscle dysfunction, and changes in microbiota compositions^[1]^. In fact, obese individuals are considered prematurely aged individuals, contributing to the phenomenon of sarcopenic obesity. Moreover, obesity is directly associated with a reduced life expectancy due to its various metabolic comorbidities. The literature on obesity and aging is well-versed, but discussion on their relationship and its underlying mechanisms is lacking. This review intends to examine the link between obesity and aging, highlighting the role of the gut microbiome [Figure 1]. Briefly, obesity and aging will be introduced, as well as their unique association and shared features. The role of the gut microbiota in obesity and aging will be discussed with associated evidence of microbial dysbiosis. A potential pathway from gut dysbiosis to adipose tissue dysfunction will be presented, exploring it as a possible underlying mechanism for obesity and aging. In addition, the bidirectional communication system between the enteric and central nervous systems, known as the gut-brain axis (GBA), will be emphasized as a potential mediator between obesity and longevity. This review also aims to present the GBA as a novel therapeutic target for obesity and aging. Specifically, the microbiome, probiotics, synbiotics and other biotic therapies may be used to beneficially modulate the gut microbiota composition, improve GI functions, and regulate GBA signaling. Biotic-based therapies will be discussed as alternatives to currently approved anti-obesity and longevity-promoting pharmacotherapies. Future perspectives on biotics as next-generation therapeutics are offered, warranting additional research. Overall, targeting the gut with microbiome-engineered therapeutics is a promising strategy for the treatment and/or management of obesity, longevity, and other chronic diseases. Recent findings will be discussed.

## THE GLOBAL BURDEN OF OBESITY: A CHRONIC, MULTIFACTORIAL DISEASE

The worldwide prevalence of obesity has tripled since 1975 and is continuing to advance^[2]^. The influence of the Western diet and sedentary lifestyle throughout the world have led obesity to become a global epidemic and economic burden. According to the World Health Organization (WHO) 2016 global estimates, 1.9 billion adults are overweight (body mass index (BMI) ≥ 25) and 650 million are considered obese (BMI ≥ 30)^[3]^. Obesity also affects 340 million adolescents and 39 million children, for a total impact of 1 billion people worldwide. Moreover, the Global Burden of Disease group stated that elevated BMI values were responsible for four million deaths in 2015^[4]^. Obesity is a chronic, relapsing, multifactorial disease characterized by an increase in body fat, white adipose tissue (WAT) dysfunction, and abnormal fat mass physical forces. The systemic consequences of obesity include adverse metabolic, biochemical, and psychosocial outcomes due to its complex underlying pathophysiology. Notably, obesity is a leading risk factor for cardiovascular disease among other cardiometabolic risk mediators and comorbidities, including insulin resistance, glucose intolerance, type 2 diabetes, hypertension, dyslipidemia, and systemic inflammation^[1]^. The fundamental cause of obesity or weight gain involves an energy imbalance between calories consumed and calories expended. However, given the complexity of the disease, obesity is further defined as a “chronic, relapsing, multi-factorial, neurobehavioral disease, wherein an increase in body fat promotes adipose tissue dysfunction and abnormal fat mass physical forces, resulting in adverse metabolic, biomechanical, and psychosocial health consequences” by the Obesity Medicine Association (OMA)^[3]^. Poor dietary habits and a sedentary lifestyle are also leading factors behind the pathogenesis and progression of obesity.

## HUMAN AGING: HALLMARKS AND IMPLICATIONS

Aging is a general biological phenomenon that affects all human beings and is characterized by a progressive loss of physiological integrity, leading to functional decline and increased risk of chronic ailments and death^[5]^. The population of older persons is one of the fastest-growing age groups, representing 10% of the global population in 2022 and expected to reach 16% by 2050, exceeding more than twice the number of children under the age of five and approximately the same as the number of children under the age of twelve^[6]^. Although aging is a natural and inevitable process, accelerated aging (i.e., when biological age exceeds chronological age) is a primary risk factor for chronic illness and mortality^[7]^. According to a recent study investigating US and UK populations, accelerated aging mediates the link between unhealthy lifestyles (i.e., physical inactivity, unhealthy BMI, unhealthy diet, smoking or drinking) and adverse health outcomes, likely via inflammation or immunity. In contrast, healthy aging is defined as developing and maintaining the functional ability that enables well-being in older age^[8]^. The study of geroscience - the intersection of basic aging biology, chronic disease, and health - has gained popularity in the last decade^[9]^. Breakthroughs in age-related research have enabled the prevention and improvement of chronic illness associated with aging, revealing key biomarkers to target aging and chronic disease. However, aging remains the single largest risk factor for heart attacks, stroke, cancer, and diabetes, among most other chronic diseases^[10]^. Age-related diseases include physical ailments - such as cancer, metabolic disease, cardiovascular disease, musculoskeletal disorders, or frailty - and cognitive impairment via neurodegenerative diseases (e.g., Alzheimer’s disease, Parkinson’s disease)^[10,11]^. Introduced by López-Otín *et al.* in 2013, the traditional hallmarks of aging are evident on molecular, cellular, and systemic levels and include genomic instability, telomere attrition, epigenetic alterations, loss of proteostasis, deregulated nutrient sensing, mitochondrial dysfunction, cellular senescence, stem cell exhaustion, and altered intercellular communication^[12]^. However, López-Otín *et al*. recently extended the list of aging hallmarks to include disabled macroautophagy, chronic inflammation, and microbial dysbiosis^[13]^. These influential additions may encourage the development of novel anti-aging strategies. Overall, individual aging hallmarks are interconnected and contribute to the progression of aging and age-associated diseases. Further research may use the identified hallmarks as biomarkers to study the aging process and anti-aging therapeutics.

## THE INTERRELATIONSHIP BETWEEN OBESITY AND AGING

### Obesity accelerates aging

As the global prevalence of obesity increases, so does the risk of dying prematurely. The association between obesity and years of life lost (YLL) has become a popular area of study in recent years. Evidence shows an increase in all-cause mortality in both overweight and obese individuals, with hazard ratios higher at younger ages than older ages and higher in men than women, across four continents^[14]^. Similarly, an Australian study showed that adults aged 20-39 years with obesity and severe obesity, compared to healthy weight adults, lose 5.6-7.6 and 8.1-10.3 years, respectively, with men losing 27.7% more life years compared to women^[15]^. Earlier research based in the US corroborates this, demonstrating that obesity shortens lifespan by up to 20 years, especially among younger adults^[16]^. Obesity also accelerates the onset of age-associated diseases, including type 2 diabetes^[17]^, cancer^[18]^, cardiovascular disease^[19]^, neurodegeneration, and cognitive decline^[20]^. Obesity and aging share several areas of overlap. A less active lifestyle, poor dietary habits, genetic predisposition, and dysregulated hormonal signaling and metabolism may cause obesity or aging^[13,21]^. In addition, the phenotype and physiological characteristics of obesity closely mimic those of aging. For example, excess reactive oxygen species (ROS) production and resulting redox imbalance, mitochondrial dysfunction, and cellular senescence are associated with obesity and aging^[22]^. Key factors that underlie obesity and aging - adipose tissue dysfunction, low-grade systemic inflammation, and disabled autophagy - will be discussed in greater detail.

### Dysfunctional adipose tissue is observed in obesity and aging

Obesity and aging share many hallmarks, such as metabolic dysregulation, insulin resistance, impaired immunity, and chronic, low-grade inflammation^[22]^. Like obesity, aging is also associated with adipose tissue dysfunction, including WAT redistribution and increased visceral adipose tissue due to reduced preadipocyte tissue differentiation^[23]^. Such changes to adipose tissue may contribute to systemic inflammation with advancing age. Adipose tissue stores energy as lipids, releasing fatty acids in response to systemic nutritional and metabolic needs, and is present in the forms of white and brown adipose tissue (BAT)^[24]^. Both types of adipose tissue are comprised of adipocytes, stem cells, preadipocytes, immune cells, fibroblasts, and endothelial cells. WAT stores energy in the form of triglycerides, while BAT expends energy via thermogenesis. Moreover, adipose tissue is divided into subcutaneous depots under the skin or visceral depots that surround internal organs. In obese individuals, visceral depots of WAT expand in size in an unhealthy manner (i.e., hypertrophy) due to increased lipid storage and in number (i.e., hyperplasia) from the proliferation of precursor cells rather than sending fat into mature adipocytes. These changes lead to WAT dysfunction and the promotion of obesity-associated metabolic complications, also leading to ectopic lipid deposition in non-adipose organs^[23]^. WAT may also undergo senescence and subsequent hypertrophy with obesity, which is also seen with aging. Aging is associated with the progression of WAT dysfunction, largely through repressed mitochondrial activity which may reduce fatty acid oxidation and lead to adipocyte hypertrophy and obesity during aging^[25]^. Accumulation of senescent cells, inflammation-inducing mechanical stress (e.g., expansion of extracellular matrix), systemic inflammation, and exogenous lipopolysaccharides (LPS) may all contribute to adipose tissue dysfunction during aging^[26]^. Of course, aging-associated susceptibility to obesity can also trigger adipose tissue inflammaging. Considering adipose tissue dysfunction is a determinant of obesity and prevalent in aged individuals, it may be a novel therapeutic target for anti-obesity and anti-aging strategies.

### Low-grade systemic inflammation as a marker of obesity and aging

Like aging, obesity is associated with a state of chronic low-grade inflammation^[23]^. As a result of weight gain or obesity, WAT undergoes a phenotypic change from lean to inflamed and dysfunctional. Under a lean status, WAT is enriched with anti-inflammatory immune cells and regulates whole-body metabolism through the secretion of adipose tissue-specific cytokines, known as adipokines (e.g., leptin, adiponectin, omentin, tumor necrosis factor-alpha (TNF)-α, interleukin (IL)-6, resistin, *etc*.)^[27]^. However, under an obese phenotype, multiple types of pro-inflammatory immune cells infiltrate WAT to trigger an inflammatory cascade and dysregulate adipokine expression. Inflamed adipocytes then secrete pro-inflammatory cytokines locally and systemically, impacting distal organs^[28]^. Sustained overnutrition maintains WAT expansion and chronic, low-grade inflammation, making obesity an inflammatory immune disease. Evidence of systemic and tissue-specific inflammation in obesity is well-established; in fact, serum inflammatory markers act as obesity biomarkers in clinical trials^[29]^. The role of inflammation in obesity is also proposed to mediate its link to other diseases, such as metabolic syndrome (MetS) and cancer^[18]^. As stated earlier, low-grade inflammation is a hallmark of aging. The concept of inflammation increasing with age, termed “inflammaging”, was introduced more than two decades ago^[12]^. It results from chronic physiological stimulation of the innate immune system, leading to increased circulating levels of pro-inflammatory cytokines and biomarkers [e.g., C-reactive protein (CRP)] and a state of chronic, low-grade inflammation. Today, inflammation is understood to be a cornerstone of many age-related diseases, including metabolic diseases^[12]^. In particular, metaflammation occurs from excessive nutrient intake or overnutrition and sustains various metabolic disorders, such as obesity and type 2 diabetes^[30]^. Overproduction of ROS is also associated with obesity, along with a decrease in antioxidants, which together lead to oxidative stress and further the complications of obesity^[22]^. A similar impact is observed with unhealthy aging, as the accumulation of ROS is responsible for many age-related functional losses. Inflammation is therefore a widespread and opportunistic target for the treatment and/or management of obesity and aging.

### Evidence of disabled autophagy in obese and aged individuals

Autophagy (i.e., macroautophagy) is a conserved biological process by which cells perform self-eating to enable the degradation of unnecessary or damaged intracellular components^[31]^. Autophagic activity maintains the orderly recycling of cellular components, protects cells against stress, and generates energy through ATP production, thereby essential for health homeostasis. The core process of autophagy is initiated by the inhibition of the mechanistic target of rapamycin (mTOR) or activation of 5’ AMP-activated protein kinase (AMPK), typically in response to stress (e.g., state of starvation) or physical activity^[32]^. mTOR and AMPK are considered master regulators of cellular metabolism, growth, and survival (e.g., autophagy pathways). However, autophagic activity declines with age and is now considered a hallmark of aging^[13]^. A reduction in autophagy-required genes and proteins (e.g., ATGs, Sirt1) in aged individuals may influence age-associated autophagy decline, although exact mechanisms remain unknown^[32]^. Inhibition of autophagy accelerates the aging process and several disease pathologies, including neurodegenerative disorders. Specifically, age-related protein aggregation may occur because of decreased autophagic activity, leading to neurodegenerative disorders, such as Alzheimer’s disease (AD) and amyotrophic lateral sclerosis (ALS). Dysfunctional autophagy is also implicated in many metabolic diseases, including obesity, insulin resistance, diabetes mellitus, and atherosclerosis^[22]^. In a healthy state, cells can normalize intracellular fat content by performing an autophagy-dependent process, known as lipophagy, to metabolize lipid droplets^[32]^. However, following excess intake of insulin and nutrients, autophagy is suppressed in skeletal muscle, adipose tissue, and the liver due to mTOR activation. Insufficient autophagy results in an unhealthy accumulation of lipid droplets, protein aggregates, and other potential autophagic substrates, leading to systemic lipotoxicity, impaired cell function, and eventually cell death. Impaired autophagy may also promote the secretion of pro-inflammatory cytokines (e.g., IL-1β) and oxidative stress. Likewise, oxidative stress can inhibit autophagy, thereby triggering endoplasmic reticulum (ER) stress and increased apoptosis in skeletal muscle and other cell types^[22]^. Recently, peroxisome proliferator-activated receptor-gamma (PPAR-γ), the master regulator of adipogenesis, was discussed as a potential modulator of autophagy in obesity^[33]^. Briefly, PPAR-γ activation increases fat storage in adipose tissue via adipogenesis, decreasing lipotoxicity in the liver and skeletal muscle. PPAR-γ stimulation may also initiate adipocyte differentiation and autophagy in fat cells, preventing adipocyte hypertrophy. However, further research is needed to determine the therapeutic potential of targeting PPAR-γ and other factors to activate autophagy for the improvement of obesity and aging.

### Sarcopenic obesity

Obesity is also accompanied by decreased muscle mass and function, as well as fatigue, due to metabolic and cellular changes to skeletal muscle from nearby increased adipose tissue^[34]^. Age-related loss of muscle mass and strength or physical performance is an aging phenomenon known as sarcopenia. The common intersection of obesity and sarcopenia led to the introduction of sarcopenic obesity, defined by a relatively low muscle mass and strength in combination with obesity. Sarcopenic obesity is mainly triggered by adipose tissue and skeletal muscle inflammation. The shared mechanisms underlying obesity and aging - such as WAT expansion, low-grade chronic inflammation, and disabled autophagy - play a part in the pathogenesis of sarcopenic obesity^[35]^. In addition, age-related reduction in muscle mass leads to weight gain in the form of fat rather than lean mass^[36]^. The deposition of fat in muscle, or intramyocellular lipids, occurs with aging and obesity, impairing muscle function through inflammation and lipotoxicity. These factors contribute to the development and progression of sarcopenic obesity, though the cellular and molecular mechanisms underlying the etiology of sarcopenic obesity remain unclear. The typical patient with sarcopenic obesity is over the age of 60 years and exhibits a sedentary lifestyle with poor nutrition^[37]^. However, sarcopenic obesity is also found in individuals as young as 20-29 years old, although its prevalence dramatically increases with age^[38]^. It is estimated that 11% of today’s global population of adults aged 60 and older have sarcopenic obesity^[39]^. Expert consensus on sarcopenic obesity was recently published, defining the disease as the co-existence of excess adiposity and low muscle mass/function^[40]^. There is currently no approved pharmacological treatment for sarcopenic obesity. Implementation of a universal definition and diagnostic criteria for sarcopenic obesity is a critical first step toward proper treatment guidelines. Moreover, sarcopenia and obesity may act synergistically to elevate the risk of cardiometabolic diseases, energy imbalance, muscular dysfunction, and, of course, mortality. For example, rates of insulin resistance and MetS in patients with sarcopenic obesity exceeded those in patients with sarcopenia alone or obesity alone^[41]^. Urgent attention and exploration of sarcopenic obesity is therefore required.

## THE GUT MICROBIOME CONNECTS OBESITY AND AGING

### The gut as an endocrine organ

The functions of the GI tract are largely regulated by gut hormones. Enteroendocrine cells (EEC) are dispersed throughout the gut epithelium, making up 1% of the GI epithelial cell population, and collectively form the largest endocrine system in the human body^[42]^. EECs modulate GI function by secreting over twenty gut regulatory peptides in response to nutrients. Ghrelin, a key hormone produced by EECs in the stomach, stimulates appetite and growth hormone (GH) release. Contrarily, glucagon-like peptide 1 (GLP-1), polypeptide YY (PYY), and cholecystokinin (CCK) are well-known satiety hormones. After ingestion of fats and carbohydrates, EECs in the ileum and colon release GLP-1 and PYY to delay the transport of nutrients from the stomach into the small intestine (i.e., gastric emptying), enabling optimal digestion and absorption of nutrients. GLP-1 also stimulates the secretion of glucose-dependent insulin, while PYY increases water and ion absorption in the colon and prevents their secretion. Produced in the small intestine, CCK also delays gastric emptying, stimulates the release of bile and pancreatic enzymes (e.g., intestinal lipase) and transmits satiety signals to the central nervous system (CNS) via vagal afferents. The GI is innervated by intrinsic neurons of the enteric nervous system (ENS) and axons of the extrinsic sympathetic, parasympathetic (e.g., vagus nerve), and visceral afferent neurons^[43]^. The anatomy of the GI enables bi-directional communication to the CNS, known as the gut-brain axis (GBA). The three main mediators of the GBA include neuronal messages carried by vagal afferents, endocrine messages carried by gut hormones, and immune messages carried by cytokines.

### Gut dysbiosis in obesity and aging

The human GI tract is home to a huge and complex microbial ecosystem made up of trillions of microorganisms, including bacteria, fungi, parasites, and viruses^[44]^. This community, known as the gut microbiota, plays an imperative role in human health and disease. A healthy gut microbiota is required for nutrient metabolism, immunomodulation, protection against pathogens and integrity of the GI tract. The majority of the microbiota belong to the gram-positive phylum *Firmicutes* (~51%) and gram-negative phylum *Bacteroidetes* (~48%), necessitating a delicate balance between them, known as the *Firmicutes* to *Bacteroidetes* (*F/B)* ratio. The remaining 1% of the gut microbiota is constituted by other less populous phyla, including *Proteobacteria*, *Actinobacteria*, *Fusobacteria*, *Spirochaetes*, *Verrucomicrobia*, and *Lentisphaerae*. It is well-known that the gut microbiota changes throughout one’s lifespan and reaches relative stability in adulthood^[45]^. However, alterations to the gut microbiota, known as microbial dysbiosis, may occur and are characterized by changes to local bacterial composition or bacterial metabolic activity^[43]^. Given its central role in the GI system, the gut microbiota is largely influenced by dietary habits. For example, a high-fat diet (HFD) or a diet rich in animal proteins can impair gut eubiosis. In fact, germ-free mice are protected against diet-induced obesity associated with a Western, high-fat and high-sugar diet, compared to mice with a gut microbiota^[46]^. Moreover, diet differs based on geographical factors, which is reflected by varying gut compositions of similar cohorts from different populations. These notions and the significance of dietary factors on the gut microbiota have been reviewed in detail previously^[47]^. Lifestyle, age, disease, cognitive function, antibiotic treatment, and numerous other factors can also modulate the microbiota, triggering dysbiosis^[48]^.

A comparison of gut composition and function associated with obesity and aging is critical for the discussion of their proposed interrelationship. Evidence of gut microbial alterations in obese individuals is well-established^[49]^. However, a dysbiotic gut has only recently been recognized as an aging hallmark^[50]^. There are general trends in literature regarding gut microbial alterations associated with obesity or aging, including a relatively higher *F/B* ratio and reduced bacterial α-diversity (i.e., richness and complexity of the microbial ecosystem)^[51-54]^. However, a clearly defined microbial profile for either obesity or aging, among most diseases, has not yet reached a consensus^[55]^. Population-based studies have been conducted to better understand microbiota shifts associated with these diseases. A cross-continental systematic review discussed gut microbiota differences in obese individuals from Europe, America, and Asia^[56]^. Briefly, obese individuals from Japan and France display a higher *F/B* ratio compared to their non-obese counterparts. Within the *Firmicutes* phylum, the abundance of *Christensenellaceae* family is significantly higher in individuals with a lean BMI compared to those with an obese BMI, according to a study performed in twin pairs from the UK. Interestingly, investigation of overweight or obese individuals with cardiovascular disease emphasized the impact of gender and BMI on the *F/B* ratio, reporting an increased *F/B* ratio correlated with BMI in women but not men. No differences in bacterial diversity were found among men and women, nor the non-stratified obese and non-obese groups. Another large cross-sectional study of obese, overweight, and healthy-weight adult Americans found a global association between BMI and microbiota richness, while observing a reduction in richness in obese women compared to healthy-weight women, which was not found in men^[57]^. Although there was no trend observed between the *F/B* ratio and BMI, several sub-taxa were associated with obesity compared to healthy-weight subjects, including enriched *Streptococcaceae*, *Lactobacillaceae*, *Actinomycetaceae*, and *Enterobacteriaceae* families and decreased *Christensenellaceae*, among other *Clostridiaceae* families. Moreover, overweight individuals showed similar gut microbial composition as obese individuals.

The impact of weight loss following dietary (i.e., energy-restricted) or surgical intervention on the gut microbiota has also been assessed. A systematic review and meta-analysis recently performed by Koutoukidis *et al.* included trials of weight loss interventions in overweight or obese adults on an international scale (i.e., 17 countries across five continents)^[58]^. Findings reported an overall increase in α-diversity with weight loss, with clear evidence for RYGB and inconsistent evidence for dietary interventions. At the phylum level, a lower *F/B* ratio and enriched levels of *Proteobacteria* and *Verrucomicrobia* following weight loss were reported, although the changes were not significant. However, the abundance of *Akkermansia*, *Bacteroides*, and *Bifidobacterium* genera increased with weight loss, while there was no evidence of changes in the presence of *Lactobacillus*. Reduced intestinal permeability with weight loss was also reported, but no change in intestinal inflammation was found. Changes to the gut microbiota with weight loss did not differ between types of intervention. In addition, differences in microbiota-related metabolites after bariatric surgery, RYGB, or sleeve gastronomy (SG) have been analyzed^[59]^. Data suggest that the metabolite p-cresol increases after SG compared to RYGB, which may be due to increased levels of *Bacteroides* with weight loss, which is one of the genera that ferments amino acids phenylalanine and tyrosine to produce p-cresol.

Gut dysbiosis in obese-aged (i.e., aged 60 and older) compared to healthy-aged older adults was recently reviewed by Tavassol *et al*. Cross-continental data revealed a decreased abundance of the *Christensenellaceae* family and a higher abundance of *Prevotella*, order *Clostridiales Incertae Sedis XIII*, *Ruminococcus* and *Staphylococcus* in obese-aged individuals^[60]^. In other studies, *Akkermansia*, *Clostridium*, *Clostridiales* and *Ruminococcus-1* were negatively associated with obesity. For individuals with metabolic syndrome, BMI and abundance of *Prevotella* were negatively associated. The authors explained the discrepancies between *Prevotella* and *Ruminococcus* genera through their association with obesity in western populations, but leanness in eastern cohorts. Similar reasoning can be applied to other inconsistencies found in literature, given the significant influence of diet and environmental factors on gut microbiota composition, as discussed above. Nevertheless, many of these findings are aligned with those from obese or aged cohorts, suggesting a similar trend in gut microbiota alterations as a result of obesity, aging, or their comorbidity.

The process of aging may disrupt gut homeostasis through a variety of factors, such as a weak immune response (e.g., via dendritic cell dysfunction), repeated exposure to antibiotics, decreased intestinal motility, and less nutrient turn-over, as well as altered diets to accommodate reduced chewing strength^[45,61,62]^. Moreover, numerous age-related diseases are associated with gut dysbiosis. A recent meta-analysis compared the gut microbiota in AD patients to that in individuals with mild cognitive impairment and healthy elders^[63]^. Findings show that individuals with AD but not cognitive impairment have reduced bacterial α-diversity compared to healthy controls. Moreover, AD patients harbor enriched levels of *Proteobacteria* and less abundance of *Firmicutes*. A trend toward decreased abundance of *Clostridiaceae* is observed in patients with mild cognitive impairment, and further pronounced in AD patients. Moreover, AD patients exhibit a mild, although not significant, increase in *Firmicutes* and *Actinobacteria*, a decrease in *Bacteroidetes*, and no change in *Proteobacteria.* Geographical differences were also found, including an increase in *Bacteroidetes* phylum in US AD cohorts, compared to Chinese AD cohorts. Another study analyzed reports of the gut microbiota of frail and non-frail older adults (i.e., ≥ 60 years) across several countries^[64]^. Compared to non-frail elders, the frail group had a lower abundance of phyla *Firmicutes* and *Verrucomicrobia*, *Bacteroides* and *Prevotella* genera, and *Faecalibacterium prausnitzii* species, as well as a higher relative abundance of genera *Bifidobacterium, Eubacterium,* and *Lactobacillus*. Interestingly, α-diversity did not differ between frail and non-frail individuals, nor did the abundance of the *Akkermansia* genus.

To understand the microbiota composition of healthy aged individuals, the microbial profiles of centenarians (i.e., individuals 100 years or older), a model of healthy aging, have been studied. Findings from different populations reveal healthy centenarians to have increased levels of health-associated bacterial species such as *Akkermansia muciniphila*, a mucin-degrading bacterium that increases mucus thickness and thus promotes intestinal integrity, compared to younger individuals or dying centenarians^[65-67]^. The *Bifidobacterium* genus is enriched in European centenarian populations, including those of Estonia and semi-supercentenarians (i.e., aged 105-109) of Italy^[66,68]^. Specifically, the *B. bifidum* species is highly abundant in Sardinian centenarians, as well as α-diversity^[65]^. *Bifidobacterium* dominates in the gut microbiota of healthy infants yet declines with age^[45]^, inferring a potential role in longevity. However, a study on Chinese centenarians revealed that the *Bifidobacterium longum* strain increases before death compared to their healthy counterparts, suggesting a negative association with lifespan^[67]^. Pang *et al.* reported similar findings on a Chinese cohort, concluding that the gut microbiota of centenarians harbors a youth-associated microbial community, including a decreasing abundance of *Proteobacteria* and an enriched abundance of *Bacteroides*^[69]^. Another study of Chinese nonagenarians (i.e., individuals aged 90-99) reported a lower *F/B* ratio, decreased abundance of *Streptococcus* genus, and increased levels of *Clostridiales* species in healthy long-living individuals, compared to non-healthy controls^[70]^. However, a US-based study identified *Bacteroides* depletion as a marker of healthy aging, suggesting its dominance in predicting decreased survival in older individuals (i.e., aged 85 and older)^[71]^. Different gut microbiota composition among separate centenarian and healthy elder populations may be due to differences in epigenetics, regional dietary habits, and other external factors that lead to region-specific microbiota profiles. Nevertheless, the gut microbial communities found in centenarians of various origins seemingly oppose those of unhealthy aged or obese cohorts.

### Alterations to gut function in obesity and aging

Along with compositional changes, the gut environment undergoes functional alterations during obesity and unhealthy aging. Physiological changes resulting from microbial dysbiosis can lead to systemic immune dysregulation. For example, a dysbiotic gut may cause an increased abundance of Gram-negative peptidoglycan bacteria, such as pathogenic *Escherichia coli*, which contain endotoxin LPS in the outer membrane. LPS can impair the intestinal epithelial lining and increase gut permeability, leading to a “leaky gut” and subsequently entering the bloodstream. Upon translocation, LPS binds to Toll-like receptor 4 (TLR4) and activates a pro-inflammatory signaling cascade via the nuclear factor-κB (NF-κB) pathway^[72]^. The resulting state of metabolic endotoxemia promotes chronic, low-grade inflammation. Translocation of LPS and other bacterial products have been associated with obesity and aging, as well as type 2 diabetes, psoriasis, and other immune-mediated diseases^[73,74]^. The gut microbiota plays a central role in both inflammaging and metaflammation through the release of LPS and other inflammatory products, contributing to crosstalk with other organs and systems^[75]^. Many studies suggest the *F/B* ratio increases with obesity and aging, as discussed above. However, a relative decrease in *Bacteroidetes* challenges the hypothesis of LPS-induced metabolic endotoxemia, as the *Bacteroidetes* phylum is the main group of Gram-negative bacteria in the gut microbiota^[55]^. Enriched levels of *Proteobacteria*, another Gram-negative phylum of which *Escherichia coli* is the dominant species, are reported in obese and aged individuals^[50,76]^. LPS derived from *E. coli* elicits an immune response and inflammatory cytokine release and enables endotoxin tolerance, whereas *Bacteroidetes* LPS inhibits immunogenicity by *E. coli* LPS^[77]^. Thus, the characteristic differences in LPS subtypes may explain metabolic endotoxemia observed in obese and aged patients, despite the apparent decrease in *Bacteroidetes* species.

As a result of a HFD, mice exhibit a hyperpermeable gut, evidenced by a decrease in trans-epithelial resistance and subsequent mucosal inflammation^[78]^. The intestinal barrier is also disrupted via restructuring of claudins, a family of tight junction (TJ) proteins, which can occur independent of diet^[79]^. These changes cause the intestinal barrier to become deregulated, contributing to systemic disruption and the obese phenotype. Barrier dysfunction may also promote dysbiosis associated with obesity or aging. Dietary fats may impair the gut mucosal layer through inhibition of Mucin 2 (*Muc2*) expression (i.e., the most important component synthesized by intestinal goblet cells)^[80]^, downregulation of TJ proteins, and induction of colonic and systemic inflammation^[81]^. Similarly, age-related decline involves localized loss of the gut mucosal barrier^[82]^. Mice deficient in *Muc2* or *Foxo1* develop gut dysbiosis, including an elevated *F/B* ratio and abundance of *Proteobacteria*^[83,84]^. Such evidence suggests that intestinal barrier dysfunction impacts gut homeostasis and may contribute to enriched levels of harmful, LPS-producing bacteria in the host. However, direct investigation into the relationship between barrier impairment and subsequent dysbiosis in obese or aged models is needed. Similar findings are reported in aged organisms; old baboons exhibit increased colonic permeability and decreased TJ protein expression (i.e., ZO-1, occludin, JAM-A), compared to young models^[85]^. The data showed a slight increase in claudin-2 in the older models, although not significant; an upregulation of claudin-2 enhances intestinal permeability^[86]^. In addition, mRNA levels of pro-inflammatory cytokines were increased (i.e., IFN-γ, IL-6, IL-1β) in the colon, indicating age-associated inflammation^[85]^. Beyond structural barrier changes, the function of gut microbes is altered in obese and aged individuals. An imbalanced intestinal microbiota contributes to obesity through energy harvest from short-chain fatty acids (SCFAs), inflammatory response driven by LPS translocation, and hormonal mechanisms that regulate appetite control^[87]^, among other factors. SCFAs also play a critical role in healthy aging, wherein their decline is associated with unhealthy aging, as evident in neurodegenerative disorders^[43]^. SCFAs (e.g., acetate, propionate, butyrate) are the product of the fermentation process by intestinal microbes on indigestible dietary fibers and are either reabsorbed by the colon to contribute to a variety of physiological processes or excreted in feces. Notably, SCFAs activate G-protein-coupled receptors 41 and 43 (GPR41/43) on enteroendocrine cells, which promote PYY and GLP-1 secretion, respectively, and increase insulin sensitivity^[88]^. SCFA receptors are also present on adipocytes, namely GPR41/43 and GPR109a, which stimulate leptin secretion upon activation, thereby suppressing insulin signaling and adipogenesis. G protein-coupled receptors are also expressed on pancreatic β cells, which can be mediated by SCFAs to modulate insulin secretion. The abundance of SCFA-producing bacteria, such as *Faecalibacterium prausnitzii*, is reduced in obese and aged gut microbiota^[82,89,90]^. Research shows a relationship between higher fecal SCFA concentrations and measures of obesity, gut permeability, metabolic dysregulation, and hypertension^[91]^, indicating a lack of absorption of SCFAs. Earlier reports also show a loss of genes for SCFA production in aged humans, compared to younger adults, as well as lower microbiota production of essential amino acids^[92]^. These alterations could play a fundamental role in the overall nutritional status of the elderly individual, leading to sarcopenia. Similarly, bile acids (BAs) are signaling molecules synthesized in hepatocytes from cholesterol and released into the colon to facilitate metabolic processes such as glucose, lipid, and energy homeostasis through the absorption of dietary lipids and vitamins. The intestinal microbiota deconjugates primary BAs into secondary BAs via the enzyme bile salt hydrolase (BSH). However, an obese gut is associated with a decrease in BSH-producing bacteria, leading to BA dysregulation. Research shows decreased microbial transformation of BA in obese mice compared to lean mice^[93]^. In addition, plasma concentrations of BA are significantly reduced with advancing age^[94]^, which may be attributed to age-related dysbiosis^[93]^. In sum, similar compositional and functional changes to the gut microbiome are evident in obesity in aging. The gut microbiome may, therefore, act as a novel target for the treatment and/or management of obesity and aging.

In summary, evidence of gut compositional and functional changes among obese and aged individuals is expansive, although it remains elusive. Based on reported data, several trends are observed among obese and aged populations that may be extrapolated for clinical use upon further investigation [Table 1]. Specifically, a higher abundance of the *Proteobacteria* genera may indicate an underlying state of metabolic endotoxemia in metabolically compromised adults. Similarly, decreases in the *Firmicutes* class *Clostridia* or the *Akkermansia* species of the *Verrucomicrobia* phyla might be evidence of an obese or accelerated aged phenotype. Signature microbial metabolites may also act as biomarkers of obesity and aging, such as increased serum LPS, fecal SCFAs, and BAs. The measurement of microbes and their metabolites depends on relatively cost-effective, minimally invasive procedures and may help in the diagnosis and treatment management of obesity, aging, and related metabolic disorders. Assessment of these microbes and metabolites may also advance the diagnosis of sarcopenic obesity. However, significant additional research is needed before the proposed biomarkers can be adopted for clinical use. Additional region-specific meta-analyses on obese and aged cohorts would be beneficial in reaching a consensus on the distinct microbiota alterations that occur with these diseases among diet and other external variables. Assessment of the gut microbial composition in obese and aged comorbid patients would also be valuable.

**Table 1:** A summary of similar alterations to gut microbiota composition and function in obese and unhealthy aged populations

**Increased abundance**	**Decreased abundance**	**Gut functional changes**
Phylum: • *Firmicutes*^[54,70]^ • *Proteobacteria*^[50,76]^ Genera: • *Enterobacteriaceae*^[52,56,82]^ • *Streptococcus*^[57,70,82]^ • *Enterococcus*^[76,82]^ Species:• *Lactobacillus*^[56,95]^ • *Escherichia coli*^[76,82]^	Phylum:• *Bacteriodetes*^[52,54]^ Family: • *Clostridiaceae*^[57,63]^ Genera: • *Prevotella*^[52,76,82]^ Species: • *Akkermansia muciniphila*^[52,54]^ • *Faecalibacterium prausnitzii*^[82,89]^ • *Bifidobacterium*^[56,82]^ • α-diversity^[52,54]^	• Decreased TJs and barrier integrity^[78,85]^ • Decreased SCFAs^[82,90]^ • Inflamed colon^[79,85]^ • Mucus degradation^[52,76,82]^ • Increased permeability^[76,82]^ • Bacterial translocation^[79,82]^

TJs: Tight junctions; SCFAs: short-chain fatty acids.

### Gut dysbiosis leads to adipocyte dysfunction

**Figure 2:**
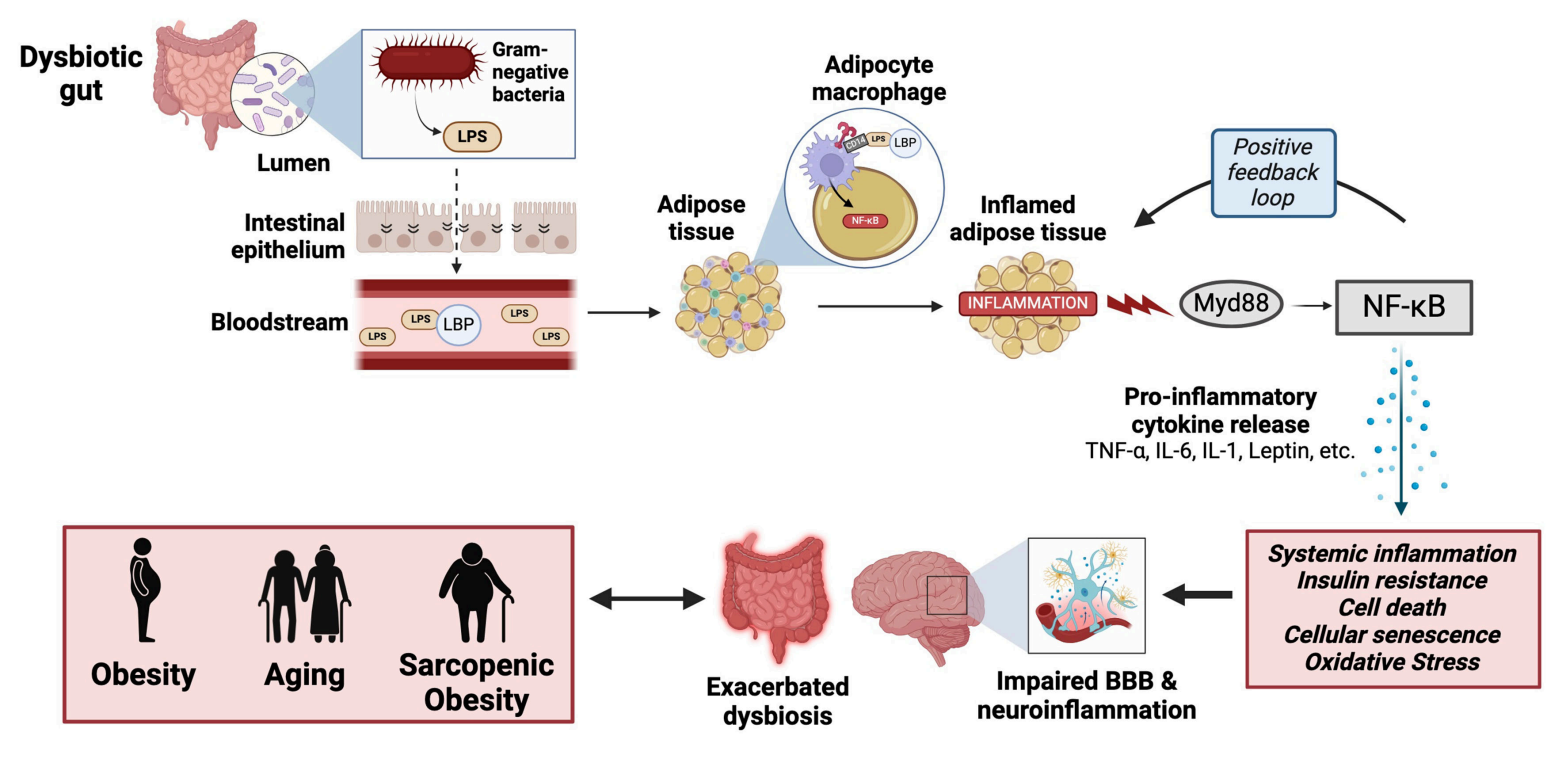
Schematic representation of the inflammatory pathway from the gut to adipose tissue and the resulting consequences on human health. LPS: Lipopolysaccharide; LBP: lipopolysaccharide binding protein; CD14: cluster of differentiation 14; NF-κB: nuclear factor kappa B.

Microbial dysbiosis plays an evident role in obesity and aging, as does altered microbiota function. As discussed, an impaired gut barrier is often associated with obesity and aging, as is the abundance of harmful bacteria, such as pathogenic *E. coli*. LPS, an abundant lipoprotein in *E. coli* and other Gram-negative bacteria, may transport across the permeable gut barrier and enter systemic circulation at high levels. Bacterial LPS may then bind to circulating LPS binding protein (LBP), eliciting an immune response. In the context of obesity or aging, macrophages may recruit LPS and infiltrate adipose tissue, especially large adipocytes, prompting inflammatory cytokine production^[96]^. Adipocytes express the required receptor for bacterial LPS, TLR4, at increased levels in the context of obesity^[97]^. Although TLR4 expression in aged and young mice is comparable, TLR4-deficient aged mice are protected from adipose tissue inflammation with aging, suggesting TLR4 to be a critical player in age-associated inflammation^[98]^. With the help of LBP and CD14, LPS eagerly binds to TLR4 receptors on adipocytes^[96]^. Activation of TLR4 triggers the MyD88-dependent pathway and subsequently NF-κB, leading to the expression of pro-inflammatory chemokines and cytokines, as well as promoting immune cell recruitment and infiltration. This behavior creates a positive feedback loop that furthers adipocyte inflammation and dysfunction, thereby promoting the obese and aged phenotypes. Clinical evidence has shown an association between LBP and central adiposity, a strong risk factor for obesity and type 2 diabetes, as well as BMI, fasting serum insulin, insulin resistance, and metabolic syndrome components, specifically obesity status^[99,100]^. There is also an inverse relationship between LBP and skeletal muscle density, potentially due to an increase in skeletal muscle adiposity^[99]^. Other reports state that obese individuals with or without comorbid diabetes have increased serum LPS levels after high-fat dietary intake compared to healthy controls, indicating an exacerbated metabolic response^[101]^. Moreover, LBP is increased in obese and non-obese aged individuals and serves as a biomarker of declining physical function and inflammation^[102]^. Increased endotoxin levels are also evident in age-related disorders and cognitive impairment, including AD and PD, among other brain pathologies^[103]^. Specifically, LPS is associated with brain amyloid uptake, suggesting it plays a mechanistic role in AD pathology^[104]^. Microbial dysbiosis and microbial translocation are therefore potential drivers of adipocyte dysfunction, a hallmark of both obesity and aging [Figure 2]. Although this idea has been previously discussed^[96]^, further mechanistic studies to help clarify the exact pathway from gut dysbiosis to adipocyte disruption would be beneficial. Such findings may identify novel therapeutic targets for obesity and aging, among other diseases associated with adipocyte dysfunction.

### The gut-brain axis: a key mediator between obesity and aging

The GBA is a complex network of neuro-immuno-endocrine mediators that connect the enteric and nervous systems. Intestinal homeostasis is therefore critical for proper brain function. When dysbiotic, the gut microbiota sends dysfunction signaling to the brain, promoting low-grade inflammation, increased oxidative stress, unbalanced energy homeostasis, and increased cellular degradation. The GBA has been implicated in several diseases involving the CNS, including mental health disorders, AD, dementia, and premature aging^[105]^. Although the exact mechanisms governing the GBA have yet to be fully identified, the involvement of the nervous, endocrine, and immune systems, as well as microbial antigens, is well-established. Briefly, the translocation of microbial antigens (e.g., LPS, SCFAs, BAs) from a permeable gut barrier may enter the CNS and pass through the blood-brain barrier, activating microglia, the resident immune cells of the CNS. As a result, neuro-inflammation is stimulated, causing a neurotoxic phenotype by activation of A1-like reactive astrocytes. In the context of neurodegenerative disease, A1-like astrocytes dominate the astrocyte population and even surround Aβ deposits in AD patients^[106,107]^. Another GBA-related mechanism regards the hypothalamic-pituitary-adrenal (HPA) axis, which utilizes the nervous and endocrine systems to regulate stress. Gut dysbiosis enables dysregulation of the HPA axis, which can accelerate and progress cognitive defects and AD^[108]^. Obesity also leads to hyperactivation and priming of the HPA axis, according to a recent study^[109]^, inferring that HPA dysfunction may link obesity and cognitive impairment. Once activated, the HPA axis triggers the stress response and release of glucocorticoids (i.e., cortisol) from the intestine to the brain. The vagus nerve also regulates the neural signaling from the gut to the brain, acting as a critical component of the GBA^[110]^. Originating from the brainstem and extending through the body, the vagus nerve innervates the GI tract with vagal afferent (80%) and efferent (20%) fibers that communicate with the brain. As the main component of the parasympathetic nervous system, the vagus nerve regulates internal organ functions, including digestion. For example, receptors of GI neurohormones (e.g., CCK, ghrelin, *etc.*) are present on vagal afferent nerve endings and either initiate or inhibit vagal afferent firing after food consumption, regulating digestive activity (e.g., gastric emptying). Interestingly, altered vagal signaling is observed with obesity or exposure to a HFD, such as an orexigenic phenotype, leading to reduced satiety or decreased sensitivity and excitability. A state of microbial dysbiosis may also alter vagal activity. In mice fed a high-fat/high-sugar or low-fat/high-sugar diet, changes to the gut microbiota (e.g., decrease in bacterial diversity, increase in *F/B* ratio) were observed, as well as reduced vagal innervation^[111]^. Additionally, an increase in the expression of pro-inflammatory cytokines, reduction in TJ protein (e.g., occludin), and an increase in cecum and serum levels of LPS were found in the mice. Further investigation of vagal activity in metabolically compromised models is warranted to determine its role in obesity and related diseases.

The vagus nerve is also proposed to play a role in aging. Dystrophic remodeling of vagal afferents in the GI may occur with age, leading to decreased vagal stimulation^[112]^. Moreover, the vagus nerve may be involved in the development of neurodegenerative diseases, such as Parkinson’s Disease (PD), through the misfolding of α-synuclein, a protein that leads to the formation of Lewy bodies that are present in neurons in PD individuals^[113]^. In AD patients, a dysbiotic gut and associated intestinal barrier disruption can induce neuroinflammation and aggravate amyloid β (Aβ) plaque formation, a known biomarker of AD. Considering the similarity between α-synuclein and Aβ pathologies, the vagus nerve has been speculated to play a role in AD similar to that of PD^[113,114]^. Thus, the link between obesity and aging may be explained, in part, by the GBA through altered vagal signaling and associated inflammation. However, additional studies are needed to clarify the role of the vagus nerve in obesity, aging, and the GBA.

## CURRENT STRATEGIES FOR TREATING OBESITY AND AGING

### Lifestyle modifications

Weight loss is achieved when an energy deficit occurs^[115]^. Energy expenditure can be increased through physical activity, while dieting may reduce energy intake. Thus, the first line of therapy for obesity regards lifestyle modifications to promote exercise and healthy dietary habits. Expert guidelines recommend an initial prescription of moderate aerobic activity of at least 150 min per week. Combining exercise with reduced caloric results in an additional 1% to 3% of body weight, with intensity of physical activity positively correlated with reduced body fat and maintained muscle mass^[116]^. Restricting calories through nutritionally balanced low-energy diets (800-1,800 kcal/day) or very low-energy diets (less than 800 kcal/day) are commonly prescribed for weight loss^[117]^. Low-calorie diets with different macronutrient profiles have demonstrated successful treatment of obesity, including ketogenic and Mediterranean diets^[118,119]^. High-protein calorie restriction diets may also be effective in reducing weight and beneficially modulating the gut microbiome in overweight or obese individuals, according to recent clinical trials^[120,121]^. A narrative review by Chao *et al*. further discusses dietary interventions for obesity and their potential mechanisms contributing to weight loss and dietary adherence^[117]^. Behavioral counseling interventions to enhance adherence to prescribed meal plans and increased physical activity are also recommended by experts, as dietary adherence is reportedly the most powerful predictor of weight loss success^[115]^. Cognitive behavioral therapy is one method that can be implemented alongside lifestyle modifications to help manage patient goals and address unhealthy behaviors, such as emotional eating^[122]^. Although behavioral modifications are recommended as first-line therapy for obesity, they often fail to achieve sustained weight loss. A meta-analysis of 27 clinical trials on obese individuals receiving nutritional weight loss interventions revealed that continuous weight gain occurs 9 months after intervention conclusion, with some patients returning to their baseline weight before one year^[123]^. Weight loss-induced physiological changes such as adaptive processes that work to prevent starvation mode^[124]^, changes in adipocyte size and function^[125]^, or inflammation from metabolic adaptation are possible mechanisms driving weight regain in obese individuals after behavior-induced weight loss^[126]^.

Lifestyle choices can be modified to achieve healthy aging and prolong lifespan. A clinical trial on healthy, biologically aging adult males (aged 50-72) successfully decreased epigenetic age by 3.23 years after 8 weeks of interventional diet (i.e., high-nutrient and plant-centered diet with fruit/vegetable powder and probiotic supplements) and lifestyle (i.e., 30 min of exercise at least 5 days/week, breathing exercises and a minimum of 7 hours of sleep)^[127]^. Like obesity, caloric restriction (e.g., 25% caloric intake) can improve aging by slowing or reversing the related molecular changes^[128]^. Metabolism can also be modulated by periodic fasting, which induces ketogenesis and increases mitochondrial DNA. Lilja *et al.* demonstrated longevity-associated changes to the gut microbiota (e.g., increased abundance of *Christensenella*) and increased expression of *Sirt1* in overweight adults (aged 23-75; average BMI of 25.93 kg/m^2^), following five days of periodic fasting^[129]^. Dietary interventions for physical and cognitive decline have also been studied in aged persons, namely the Dietary Approaches to Stop Hypertension (DASH) diet and the Mediterranean-DASH Intervention for Neurodegenerative Delay (MIND) diet^[130]^. These specific diets are rich in omega-3 fatty acids, antioxidants, and polyphenols, exerting anti-inflammatory and neuroprotective benefits. Studies of the DASH diet report significant reductions in blood pressure and an increased effect correlated with sodium intake compared to a normal diet, although the impact on elder individuals is less than on younger individuals^[131]^. Moreover, a combination of the MIND diet and aerobic exercise further improves cognitive function in obese elderly women and may substitute for sex hormone deficiency in postmenopausal women^[132]^. A long-term study of the MIND diet on brain function in overweight elders at risk for dementia is ongoing and may provide mechanistic evidence of the diet’s effects^[133]^. However, aging individuals may face societal and health service obstacles when modifying their lifestyles. Toward this end, governmental policies have been recommended by the WHO to designate the period from 2020 to 2030 as the “Decade of Healthy Ageing”^[8]^. The policies regard global data collection on the health status of older people, conducting research on healthy aging, increasing accessibility to healthcare, and establishing integrated long-term care services. Other efforts to reduce age discrimination and provide age-friendly cities and communities are also discussed. Similarly, the UK government recently released a consensus statement on healthy aging, which includes a discussion on preventative healthcare, opportunities for older adults to contribute to society, and the promotion of active and independent lifestyles^[134]^. Such policies will help create environments and promote behavior that fosters healthy aging, similar to what is implemented in blue zone populations^[135,136]^. Societal efforts to improve the lifestyle of elder individuals are encouraging and imperative for the rising aging population.

### Biological agents for the treatment of obesity and aging

Biological therapies, represented by monoclonal antibodies (e.g., anti-TNF-α agents), have been developed to neutralize pro-inflammatory cytokines for a variety of immune-mediated inflammatory diseases (e.g., rheumatoid arthritis, plaque psoriasis, ulcerative colitis, *etc*.)^[137]^. Considering the immune-related dysfunction associated with obesity and aging, the use of biological agents for their treatments is a promising idea. In aging mice, treatment with a TNF-α inhibitor (i.e., etanercept) led to significantly improved muscle function and lifespan, suggestive of therapeutic potential against sarcopenia^[138]^. In the context of aging, Chimeric antigen receptor (CAR) T-cells can be used to target upregulated, age-associated proteins (e.g., uPAR or β-galactosidase) on senescent cells to safely and effectively eliminate senescent cells^[139]^. This groundbreaking study also found uPAR CAR T cells to be therapeutically active against diet-induced obesity by promoting weight loss and improving glucose homeostasis. However, direct investigation into biological agents for the treatment of obesity is lacking. Many studies have considered the impact of such therapies on obesity as a comorbidity of psoriasis, a disease commonly treated with biologics. For example, a recent study compared the use of biological agents and conventional treatments for obese and non-obese psoriasis patients^[140]^. Findings showed successful treatment of psoriasis by IL-17 and IL-23 inhibitors in obese patients, which are aligned with previous reports^[141]^. On the contrary, TNF-α blockers are not recommended for obese patients, as they are reportedly associated with an increase in body weight and BMI^[142]^. As obesity and psoriasis share remarkably similar underlying mechanisms, anti-psoriatic therapies may also be effective in treating obesity^[143]^. However, research is significantly lacking in this area. Pre-clinical and clinical studies are needed to validate the use of new-generation biological agents for obesity and aging. Given their favorable safety profiles and minimal adverse effects, biological agents may act as alternative therapies to pharmacotherapies based on significant findings.

### Pharmacological treatment and management of obesity and aging

For individuals who have an inadequate response to lifestyle interventions, experts recommend using pharmacotherapy as an adjunct to lifestyle intervention^[144]^. Current U.S. Food and Drug Administration (FDA)-approved anti-obesity medications target peripheral and central pathways to reduce appetite and increase satiety^[145,146]^; however, they are limited by reported side effects [Table 2]. For severely obese (BMI ≥ 40) individuals, bariatric surgery and implanted devices are available, though they are more invasive and expensive than medications. Although new drugs are undergoing FDA consideration (e.g., Tirzepatide), the mechanisms of action are similar to the currently approved drugs, leading to similar safety profiles. Other medications are approved for rare obesity subtypes, as opposed to general obesity, such as Metreleptin for congenital or acquired generalized lipodystrophy and Setmelanotide for individuals with genetic variants of obesity (e.g., proopiomelanocortin, proprotein convertase subtilisin/kexin type 1, leptin receptor). In addition, some drugs share a mechanism of action with weight-loss therapies but are approved for other indications. For example, semaglutide is an injectable drug that mimics the action of GLP-1. Semaglutide is approved for weight loss as Wegovy®^[145]^, as well as for the treatment of type 2 diabetes as Ozempic®, with the two drugs differing in dosage, administration, and indication^[147]^. However, the off-label use of Ozempic® for weight loss has gained public notoriety^[148]^. Despite the numerous available options, there is a great demand for novel anti-obesity therapies with revised therapeutic targets, superior efficacy and minimal side effects compared to currently approved agents.

**Table 2:** A summary of current FDA-approved pharmacotherapies for obesity

**Name of drug**	**Mechanism(s) of action**	**Limitations**
Phentermine	Activates the sympathetic nervous system to promote release of norepinephrine, dopamine and serotonin, suppressing appetite and increasing the breakdown of stored fat for energy^[145]^	Hypertension, insomnia, constipation, dry mouth, valvular heart disease, withdrawal effects, risk of abuse and dependence, approved for short-term use only, cannot use with history of CVD, indicated as a monotherapy
Diethylpropion	Suppresses appetite and increases satiety by promoting high levels of catecholamines, dopamine and norepinephrine^[144]^	Hypertension, insomnia, constipation, dry mouth, valvular heart disease, abuse potential, epilepsy, approved for short-term use only, cannot use with history of CVD, indicated as a monotherapy
Orlistat	Decreases GI absorption of fat^[145]^	Fatty/oily stool, fecal urgency, increased defecation, fecal incontinence, reduced absorption of fat-soluble vitamins
Phentermine-topiramate	Stimulates the sympathetic nervous system and inhibits fatty acid synthesis^[145]^	Numbness, dizziness, dry mouth, constipation, altered taste perception, insomnia, anxiety
Bupropion-naltrexone	Increases dopamine and norepinephrine levels to suppress appetite and food intake^[145]^	Nausea and vomiting, constipation, headache, dizziness, insomnia, dry mouth
Liraglutide	GLP-1 receptor agonist^[145]^; Increases satiety and delays gastric emptying	Nausea and vomiting, abdominal pain, increased heart rate, thyroid cancer risk, hypoglycemia, infections
Gelesis-100	Superabsorbent hydrogel particles of a cellulose-citric acid matrix that increases volume and firmness of meals^[146]^	Abdominal distension, infrequent bowel movements, indigestion
Semaglutide	GLP-1 receptor agonist^[145]^; Reduces food cravings and energy intake	Nausea and vomiting, diarrhea, constipation, risk of hepatobiliary and gallbladder disorders, increased heart rate

FDA: Food and Drug Administration; CVD: cardiovascular disease; GLP-1: glucagon-like peptide-1; GI: gastrointestinal.

Anti-aging therapeutics, or geroprotective agents, extend lifespan, delay or improve aging hallmarks to a youth-like state, improve the overall quality of life, delay the progression of age-related diseases and/or increase resistance to adverse factors^[149]^. These agents should also exhibit low toxicity and have a conservative target and mechanism of action. Common aging targets include AMPK, the sirtuin family of proteins [e.g., Sirtuin 1 (Sirt1)], nicotinamide phosphor-ribosylltransferase (NAMPT), phosphatidylinositol-3-kinase (P13K)/Akt, mTOR, cyclic adenosine phosphate-dependent protein kinase (PKA), Forkhead Box O (*Foxo*), and insulin-like growth factor (IGF). Senolytic agents are another class of anti-aging therapies, which kill senescent cells to reduce cellular senescence. Many currently approved FDA drugs target mechanisms underlying aging, although they are indicated for other diseases. Most notably, the antidiabetic drug metformin has been proposed to be a promising anti-aging candidate^[150]^. A systematic analysis revealed the association between metformin and decreased all-cause mortality and suggested the drug may also extend lifespan^[151]^. Other evidence shows increased autophagy and improved mitochondrial function following treatment with metformin, ameliorating age-associated inflammation. Other FDA-approved drugs also hold promise as novel anti-aging candidates, although limited by adverse side effects [Table 3].

**Table 3:** Examples of current FDA-approved pharmacotherapies with anti-aging potential

**Name of drug**	**FDA-approved indication(s) of use**	**Mechanism(s) of action**	**Evidence of anti-aging potential**	**Limitations**
Metformin hydrochloride	Type 2 diabetes mellitus in adults and children as an adjunct to diet and exercise^[152]^	Antihyperglycemic, decreases gluconeogenesis, reduces intestinal absorption of glucose, improves insulin sensitivity and activates AMPK^[153]^	*PCK1* gene upregulation, *PARP1* gene downregulation and promotion of pyruvate metabolism^[154]^	Lactic acidosis, renal impairment, hypoglycemia, diarrhea, nausea and vomiting, indigestion, abdominal discomfort and/or headache^[152]^
Sirolimus	Prophylactic for organ rejection in renal transplant patients^[155]^	Inhibits T-lymphocyte activation and proliferation, antibody production and mTOR activation to suppress the immune system^[152]^	Extends lifespan, increases autophagy, reduces age-related gut pathologies^[156]^ and reduces oxidative stress^[157]^	Hyperlipidemia, renal dysfunction, hypertension, abdominal pain, nausea, headache, myalgia and/or hypercholesterolemia^[155]^
N-acetylcysteine	Potential hepatotoxic dose of APAP and abnormal mucous secretion (e.g., bronchitis)^[158]^	Promotes glutathione production to detoxify liver, breaks up mucus, scavenges free radicals and increases ATP production^[158]^	Increases lifespan, attenuates senescence and suppresses mTOR pathway^[159]^	Nausea, vomiting, diarrhea, flatus, gastroesophageal reflux and/or anaphylactic reaction (via IV)^[158]^
Dasatinib	Subtypes of leukemia in adults with resistance or intolerance to prior therapy^[160]^	Inhibits kinases and prevents the proliferation of leukemia cells^[160]^	When combined with quercetin, dasatinib (D+Q) acts as a senolytic, increases lifespan and ameliorates premature frailty^[161]^	Thrombocytopenia, neutropenia, anemia, platelet dysfunction, fluid retention, cardiac ventricular repolarization, gastrointestinal issues (diarrhea, nausea, vomiting, *etc*.), bleeding events and/or infection^[160]^

FDA: Food and Drug Administration; PCK1: phosphoenolpyruvate carboxykinase 1; PARP1: poly [ADP-ribose] polymerase 1; AMPK: 5’ AMP-activated protein kinase; mTOR: mammalian target of rapamycin; APAP: acetaminophen; IV: intravenous.

## TARGETING THE GUT MICROBIOTA WITH BIOTICS TO IMPROVE OBESITY AND AGING

The limitations of current pharmacotherapies necessitate the development of novel therapeutics for obesity and aging. Targeting the gut using biotics to manipulate the microbiome and GI environment is an attractive option to restore microbial homeostasis and host health. Biotics represent material derived from living, or once living, organisms and can exist in many forms, namely prebiotics, probiotics, synbiotics, and postbiotics. Simply, prebiotics are food components that benefit the gut microbiota, while probiotics are live microorganisms that elicit benefits to the host and synbiotics are the synergistic combination of probiotics and prebiotics^[162]^. The newest biotic form, postbiotics, represents substances released or produced by microbiota metabolism that benefit the gut microbiota and the host. Many biotics exert beneficial properties for human health that may improve obesity and promote healthy aging.

### Prebiotics

Intake of dietary fiber plays a beneficial role in gut health. Prebiotics are non-digestible fermented dietary fibers that selectively stimulate the growth of beneficial probiotics to improve host health^[163]^. The degradation of prebiotics by the gut microbiota allows beneficial microbes to obtain survival energy and release fermentation products such as SCFAs and other metabolites that influence a variety of metabolic effectors^[164,165]^. There are several types of prebiotics, including fructans and oligosaccharides, which naturally occur in dietary food products such as berries, asparagus, garlic, onion, chicory, honey, and wheat, among others. Dietary functional oligosaccharides, such as galacto-oligosaccharides (GOS), are among the most studied prebiotics for human health. Previous research has demonstrated the ability of GOS to reduce weight gain and hyperplasia and hypertrophy of adipose tissue in diet-induced obese rats to the same extent as Orlistat. Clinical evidence also suggests that supplementation of GOS leads to an increased abundance of *Bifiobacterium* strains in fecal samples of obese adults^[166]^. In aged mice, dietary GOS had significant impacts on the gut microbiome compared to age-matched controls, increasing the abundance of beneficial bacteria and reducing intestinal permeability and colon inflammation^[167]^. Interestingly, however, GOS supplementation did not have a bifidogenic effect. Other oligosaccharides have also demonstrated the ability to regulate glucose control and reduce serum lipid levels^[168]^, while studies show *Drosophila melanogaster* lifespan extension, increased antioxidant capacity, beneficial gut modulation, and improved intestinal immunity (i.e., via upregulated *IMD* gene expression) following supplementation with marine agar oligosaccharide^[169]^. Moreover, quercetin is one of the most well-studied dietary flavonoids and is present in many vegetables, fruits, tea, and red wine^[170]^. Previously, quercetin has been demonstrated to prevent HFD-induced obesity in mice, possibly mediated by lipogenesis regulation and MAPK pathway inhibition^[171]^. As presented in Table 2, quercetin is also considered a senolytic agent that may play a key role in the treatment of aging. The anti-inflammatory and anti-oxidative properties of quercetin are well-researched, although the mechanisms remain largely unknown. Recent research suggests Sirt1 as a potential mechanism for its anti-aging benefits^[172]^; however, many studies on quercetin discuss the combination therapy with Dasatinib (i.e., D+Q)^[173]^, leaving the effects of quercetin alone largely unknown. The extensive evidence of D+Q has led to the pursuit of clinical trials for its use in aging diseases, including AD^[174]^. Additionally, investigation on quercetin’s gut modulatory abilities is promising, with recent research showing amelioration of gut microbial dysbiosis and improved gut barrier function in antibiotic-treated mice and abdominal obesity-induced models^[175,176]^. Findings also reveal a positive effect of quercetin treatment on locomotor activity and oxygen consumption, inferring therapeutic potential against neuronal damage^[175]^.

Overall, the use of prebiotics as therapeutic interventions is gaining popularity. Clinical trials have investigated components that are found in the everyday diet. For example, Xu *et al*. investigated the effects of aged garlic extract consumption in adults with obesity for six weeks^[29]^, but gut microbiota composition was not analyzed. Serum inflammatory markers (e.g., IL-6, TNF-α) were significantly reduced in the treated participants, compared to those given placebo capsules, while circulating immune cells (e.g., γδ-T cells) increased, inferring fewer immune cells were present in adipose tissue. However, levels of CRP and adipokines (i.e., leptin and adiponectin) did not change in response to the intervention. Green tea extract, epigallocatechin gallate (EGCG), has also been studied for anti-inflammatory, anti-obesogenic, and anti-aging properties. A recent pre-clinical trial found that EGCG extends lifespan in high-fat-fed obese rats, while suppressing oxidative stress and activating fatty acid and cholesterol metabolism^[177]^. In addition, previous clinical studies have reported that EGCG reduces low-density lipoprotein (LDL) cholesterol and increases leptin levels in overweight and obese women after six-week supplementation^[178]^. Similarly, ellagitannins are bioactive polyphenols present in certain fruits, nuts, and seeds (e.g., pomegranate)^[179]^. Once ingested, ellagitannins are metabolized by gut bacteria to produce urolithin A, a natural compound and potential anti-aging agent that promotes mitophagy and mitochondrial function. Although evidence is growing for the use of prebiotic, natural compounds for obesity and healthy aging, additional research is needed. Future work may optimize the therapeutic effects of these prebiotics through dosage studies, methods of delivery (e.g., capsule, powder), and treatment period. Overall, various prebiotics can be used to improve obesity and aging markers. A summary of the abovementioned findings from pre-clinical studies and human trials is presented in Table 4.

**Table 4:** Pre-clinical and clinical evidence of prebiotics for the treatment of obesity or aging

**Agent**	**Disease of interest**	**Model investigated**	**Key findings**	**Ref.**
Galacto-oligosaccharides	Obesity	HFD-induced obese male Sprague Dawley rats	↓ Weight gain and WAT dysfunction, serum total cholesterol, triglycerides, HDL-C and LDL-C	Kong *et al.*^[180]^
	Obesity	Obese men and women (aged 18-65)	↑ *Bifidobacteria*	Krumbeck *et al.*^[181]^
	Obesity	Obese prediabetic men and women (aged 45-70)	↑ *Bifidobacterium* species	Canfora *et al.*^[166]^
	Aging	Aged female C57BL/6J SPF mice	↑ *Bacteroides, Akkermansia, Lactobacillus,* α-diversity; ↓ *Clostridium*, intestinal permeability, colonic TNF-α expression; No impact on *Bifidobacteria*	Arnold *et al.*^[167]^
Marine agar oligosaccharide	Aging	Male *Drosophila melanogaster*	↑ *Proteobacteria*, lifespan, antioxidant activity, upregulated *IMD* gene expression; ↓ *Firmicutes*	Ma *et al.*^[169]^
Quercetin	Obesity	HFD-induced obese male C57BL/6J SPF mice	↓ Body weight gain, total cholesterol, triglycerides, free cholesterol, total lipids, lipogenic gene expression	Jung *et al.*^[171]^
	Abdominal obesity	MSG-induced obese male C57BL/6J mice	↑ *Bacteroidetes*, lipid oxidation, locomotor activity, *Muc2*, *ZO-1* ↓ *Firmicutes*, body weight gain, inguinal and epididymal fat mass and adipocyte size, lipid deposition	Zhao *et al.*^[175]^
Dasatinib + quercetin	Aging	Aged female BALB/c mice	↑ *Verrucomicrobia* (i.e., *Akkermansia)*, Gram-positive taxa ↓ *Firmicutes*, intestinal senescence and inflammatory gene expression	Saccon *et al*.^[173]^
Aged garlic extract	Obesity	Obese men and women (aged 25-65)	↑ Circulating γδ-T cells ↓ Serum IL-6 and TNF-α	Xu *et al*.^[29]^
Epigallocatechin gallate	Obesity	HFD-induced obese male SPF Wistar rats	↑ Lifespan ↓ Body weight, serum free fatty acid, disease pathology, frailty, serum glucose, insulin, total cholesterol, triglycerides, LDL-C, IL-6, TNF- α, ROS	Yuan *et al*.^[177]^
	Obesity	Overweight and obese women (aged 18-65)	↑ Leptin ↓ LDL-C No impact on body weight, total cholesterol, triglycerides or HDL-C	Huang *et al*.^[178]^

HFD: High-fat diet; WAT: white adipose tissue; HDL-C: high-density lipoprotein cholesterol; LDL-C: low-density lipoprotein cholesterol; SPF: specific pathogen-free; TNF-α: tumor necrosis factor-alpha; IMD: immune deficiency; MSG: monosodium glutamate; muc2: mucin 2; ZO-1: zonula occluden-1; γδ-T: gamma delta T; IL-6: interleukin-6; ROS: reactive oxygen species.

### Probiotics

Probiotics are live microorganisms that elicit health benefits to the host when administered in adequate amounts^[182]^. Probiotics have a multitude of health benefits including, but not limited to, producing SCFAs, synthesizing vitamins, lowering gut pH, enhancing immune response, and exhibiting antimicrobial activity against pathogenic species^[183]^. Most notably, probiotics can modulate the gut microbiota by increasing the abundance of beneficial species when regularly consumed^[184]^. Probiotics are available in the form of functional food products (e.g., yogurt, cheese, kefir), as well as dietary supplements. The most widely used strains of probiotics include those of the *Lactobacillus* and *Bifidobacterium* genera. Thus, there is a multitude of evidence in support of probiotic therapies for obesity and aging.

#### Lactobacillus and Bifidobacterium strains improve obesity and aging in experimental models

Supplementation with various probiotic strains has been studied for its effects on obesity features. Similarly, the term “gerobiotics” was recently proposed by Tsai *et al.* to classify probiotic strains that can attenuate fundamental mechanisms of aging, reduce the physiological aging process, and extend the health span of the host^[185]^. Thus, various *Lactobacillus* strains have been investigated as obesity and aging therapeutics. *Lactobacillus fermentum* is a well-studied probiotic with evidence of reducing metabolic disease markers, including insulin resistance, hyperinsulinemia, and hypercholesteremia, *in vivo*^[186]^. Other research revealed supplementation with *L. fermentum* NCIMB 5221, as well as a probiotic formulation (*B. longum* spp. *infantis* NCIMB 702255, *L. fermentum* NCIMB 5221, and *Lactobacillus plantarum* NCIMB 8826) rescues weight gain in high-fat-fed, diet-induced obese *Drosophila*^[187]^. The individual probiotics also had positive impacts on total weight, glucose and triglyceride levels. These effects were closely mimicked in diet-induced diabetic *Drosophila* in the same study, inferring the potential of these probiotics and their combination to simultaneously treat multiple metabolic disorders with shared pathophysiology. A microencapsulated probiotic blend (*L. rhamnosus* NCIMB 6375, *L. plantarum* NCIMB 8826 and *L. fermentum* NCIMB 5221) was also studied for its effects on HFD-induced MetS hamsters and resulted in favorable alterations to the gut microbiota through decreasing the *F/B* ratio^[188]^. Although there was no impact on high-density lipoprotein (HDL) cholesterol, the probiotic-treated group showed improved metabolic regulation via reduced serum total cholesterol, LDL-cholesterol and triglyceride levels, compared to controls. Kimchi-derived *L. plantarum* Ln4 is another strain that reduces weight gain in HFD-fed mice, improves metabolic markers (e.g., reduces total triglycerides and insulin resistance, improves glucose tolerance), and decreases inflammatory adipokines (e.g., CRP, MCP-1) in WAT through immunomodulation^[189]^. The parent strain of *L. plantarum* NCIMB 8826, known as *L. plantarum* WCFS1, protects age-associated mucosal barrier decline in mice subjected to accelerated aging^[190]^. Another subspecies of *L. plantarum*, TWK10, demonstrates beneficial microbial modulatory properties by increasing SCFA-producing species in mice after its supplementation^[191]^. Moreover, age-associated loss of muscle strength, bone mass and cognitive decline was prevented in mice treated with *L. plantarum* TWK10. *L. acidophilus* has also been studied for its impact on obesity in HFD-fed mice^[192]^. Findings reveal that *L. acidophilus* reduces body weight and fat mass, activates BAT, and improves metabolic activity (e.g., energy, glucose and lipid metabolism). Obesity-induced inflammation (e.g., TNF-α, IL-1β and IFN-γ), as well as TLR4 protein expression and endotoxemia status, was also reduced after 12-week supplementation with *L. acidophilus* through NF-κB inhibition. Supplementation with *L. acidophilus* successfully resolved obesity-induced gut dysbiosis in the mice by increasing the *F/B* ratio and decreasing the abundance of *Faecalibaculum* and *Bifidobacterium,* for example. Previous literature has discussed the beneficial modulation of the gut microbiota in obese models in more detail^[193]^.

*L. reuteri* is another bacterial species with promising potential for the treatment of obesity. Recent findings reveal the anti-inflammatory activity of *L. reuteri* LM1071 in LPS-stimulated immune cells^[194]^. Another study found that the *L. reuteri* stress protein GroEL inhibits pro-inflammatory cytokines and promotes anti-inflammatory activity in human macrophages, reduces LPS-induced colonic inflammation in human biopsies, and inhibits dextran sulfate sodium (DSS)-induced colitis in murine models, likely through TLR4 signaling^[195]^. In addition to immunomodulatory properties, *L. reuteri* produces antimicrobial molecules and is able to strengthen the gut barrier to prevent microbial translocation^[196]^. Earlier research showed reduced cholesterol and increased Vitamin D serum levels in hypercholesterolemic adults after a nine-week supplementation with *L. reuteri* NCIMB 30242^[197]^, while current, ongoing research is investigating the impact of *L. reuteri* V3401 on plasma LPS levels, microbiota composition, and disease biomarkers in obese patients diagnosed with MetS^[198]^. However, other evidence reveals that *L. reuteri* may be associated with obesity and correlated with a higher BMI^[199]^. Further investigation may compare various subspecies of *L. reuteri* and determine their mechanisms of action in relation to obesity. Other strains of *Lactobacillus* have been discussed as potential agents for weight and fat mass loss in overweight and obese individuals, such as *L. casei*, *L. rhamnosus*, and *L. curvatus*, as well as their combination with other *Lactobacillus* strains or strains of the *Bifidobacterium* genera^[200]^.

*L. casei* Shirota, a probiotic reported to improve muscle function in young adult mice, was recently investigated in aged SAMP8 mice for its effects on age-related sarcopenia^[201]^. Findings revealed that 12-week supplementation with *L. casei* improves age-related decline in muscle mass and strength, decreases inflammation and oxidative stress, recovers loss of SCFAs, and enriches the gut microbiota with genera positively correlated with muscle ability, mitochondrial function, and anti-inflammatory cytokine, IL-10. As observed with the other species, the effects of *L. casei* may be attributed to the gut-muscle axis, which refers to the impact of the gut microbiota on muscle mass and function through inflammation and SCFA levels. *L. paracasei* also holds promise as an anti-aging probiotic. A recent study reported that *L. paracasei* PS23 improves cognitive function in aging mice, including memory deficits, age-related motor impairment, and anxiety behavior^[202]^. The mechanisms of *L. paracasei* PS23 were attributed to an increase in serotonin levels, antioxidant enzyme production, and beneficial gut modulation, such as an increase in *Dubosiella*, a potential anti-inflammatory species. Chen *et al.* also explored the effects of *L. paracasei* PS23 on sarcopenia and concluded that this strain attenuates age-related loss of muscle mass and strength by enhancing mitochondrial function, reducing inflammation, and improving antioxidant enzyme levels in muscle tissue^[203]^. Similarly, long-term (43-week) supplementation with *L. paracasei* K71 led to enhanced cognition and upregulated BDNF expression in senescence-accelerated mouse prone 8 (SAMP8)^[204]^. However, serotonin levels did not alter in response to treatment, compared to control, and the study did not assess gut microbial composition.

Age-related decline in immunity may be improved by probiotic supplementation. A clinical trial conducted in 2013 evaluated *L. delbrueckii* subsp. *bulgaricus* 8481 for immunomodulatory properties in elderly individuals and found an improved immune risk profile, slowed aging of T cell subpopulations, and decreased systemic levels of the pro-inflammatory cytokine, IL-8^[205]^. However, the benefits of the probiotic supplementation ceased within six months of stopping probiotic intake, suggesting that continual supplementation is required for sustained effects. Moreover, literature lacks recent clinical studies on *L. delbrueckii* or similar probiotic strains. The use of *Lactobacillus* in delaying or improving the aging process and its hallmarks is promising, yet requires additional research given the complex nature of aging and the strain-specific effects of the probiotics.

*Bifidobacterium* is a well-studied genus within the *Actinobacteria* phylum and has been studied for its health benefits in the treatment and prevention of several diseases, alongside *Lactobacillus*^[206]^. Certain *Bifidobacterium* strains have gained popularity recently, including *B. bifidum*, for amelioration of DSS-induced colitis by improving the gut barrier and inhibiting NF-κB activity^[207]^. When combined with *Lactobacillus*, *B. bifidum* also demonstrates the potential to treat cardiovascular disease through immunomodulatory properties^[208]^. A recent study showed that supplementation with *B. longum* spp. *infantis* NCIMB 702255 decreased body weight gain in diet-induced obese *Drosophila* and reduced total triglyceride and total glucose levels, compared to control^[187]^. This *Bifidobacterium* strain also reduced elevations of fatty acid oxidation genes, including acetyl Co-A carboxylase, fatty acid synthase, and phosphoenolpyruvate carboxykinase. In another study, a significant reduction in body weight gain and fat accumulation, as well as an improved plasma lipid profile, were observed in HFD-induced obese mice supplemented with *B. bifidum* DS0908 and *B. longum* DS0950, indicating the potential of *Bifidobacterium* to alleviate obesity markers^[209]^. In addition, *B. pseudocatenulatum* was recently proposed as a potential beneficial bacterium for neural and immune function improvement, based on 16S rRNA sequencing and metagenomics data from 32 longevity families of three generations (i.e., centenarians, elders, and young persons)^[210]^. The data revealed other species to have potential associations with aging, namely *Desulfovibrio piger*, *Gordonibacter pamelaeae*, *Odoribacter splanchnicus*, and *Ruminococcaceae bacterium* D5. Future studies can investigate these species for gerobiotic potential.

#### Investigation of anti-aging and obesogenic probiotics in human trials

The demonstrated efficacy of probiotics in experimental models has led to their investigation in humans. Recent clinical trials have reported excellent patient outcomes for the treatment of obesity following probiotic treatment [Table 5]^[211-214]^. The primary outcomes for these trials include improvement of various obesity markers, such as reduction in body weight and BMI, as well as improved lipid profiles. Changes to the gut microbiota are also assessed following the treatment period to identify treatment-induced shifts in composition. Clinical trials have also reported the efficacy of probiotics in aged populations [Table 5]^[215-220]^, with many studies investigating elders with mild cognitive impairment (MCI). MCI is a common precursor to AD, dementia, and other age-related diseases, such as frailty^[221]^. Alongside analyzing gut microbiota composition, many of the studies on aging evaluate cognition with validated assessment tools and measure serum brain-derived neurotrophic factor (BDNF). BDNF is a neurotransmitter modulator that plays a key role in neuroplasticity and protects against neuroinflammation and neuronal apoptosis; decreased levels of BDNF are associated with AD and mild cognitive impairment^[217]^. However, studies focused on improving physical ailments of aging as well as longevity or lifespan with probiotics are lacking.

**Table 5:** Recent double-blinded randomized controlled clinical trials on probiotic therapies for obesity and aging

	**Probiotic**	**Form**	**Population**	***N***	**Dose**	**Treatment period**	**Therapeutic effect(s)**	**Key gut microbiota shifts**	**Ref.**
Obesity	*Lactobacillus curvatus* HY7601 and *Lactobacillus plantarum* KY1032	Capsule	Overweight and obese males and females in Korea (aged 19-65; BMI 23-35 kg/m^2^)	59	1 × 10^10^ CFU/day	3 months	• Reduced body weight, BMI, waist circumference, body fat mass, lean body mass and visceral fat area • Decreased leptin and increased adiponectin	Increased: *Bifidobacteriaceae*, *Akkermansciaceae*, α-diversity Decreased: *Prevotellaceae*, *Selenomonadaceae*, *Oscillospiraceae*	Mo *et al.* (2022)^[211]^
	Kimchi-derived *Lactobacillus plantarum* K50	Capsule	Obese men and women in Korea (aged 20-65; BMI 25-30 kg/m^2^)	81	4 × 10^9^ CFU/day	3 months	• Decreased total cholesterol and triglycerides • Decreased leptin • Nonsignificant decrease in waist circumference; no change in body weight or BMI	Increased: *Lactobacillus plantarum* *Enterococcus* Decreased: *Actinobacteria*	Sohn *et al.* (2022)^[212]^
	Milk-derived *Lactobacillus plantarum* Dad-13	Powder	Overweight men and women in Indonesia (aged 35-65; BMI ≥ 25 kg/m^2^)	60	2 × 10^9^ CFU/day	3 months	• Decreased body weight and BMI • No change in SCFAs or lipid profile	Increased: *Bacteroidetes*, *Prevotella* Decreased: *Firmicutes*	Rahayu *et al.* (2021)^[213]^
	*Lactobacillus acidophilus* NCIMB 30157, *Lactobacillus acidophilus* NCIMB 30156, *Lactobacillus plantarum* NCIMB 30280, *Bifidobacterium bifidum* NCIMB 30153 and *Bifidobacterium animalis* subsp. *Lactis* NCIMB 30172	Capsule	Overweight men and women in Bulgaria (aged 45-65; BMI 25-29.9 kg/m^2^)	70	5 × 10^10^ CFU/day	9 months	• Clinical weight loss • Reduced BMI, waist and hip circumference	N/A	Michael *et al*. (2021)^[214]^
Aging	*Lactobacillus rhamnosus* GG	Capsule	Men and women with MCI in USA (aged 52-75)	169	1 × 10^10^ CFU/day	3 months	• Improved cognitive performance	Increased: *Lactobacillus* Decreased: *Prevotella*	Aljumaah *et al.* (2022)^[215]^
	*Lactobacillus plantarum* C29-fermented soybean	Capsule	Men and women with MCI in Korea (aged 55-85)	92	1 × 10^10^ CFU/day	3 months	• Improved cognition • Increased serum BDNF levels	Increased: *Lactobacillus*	Hwang *et al*. (2019)^[216]^
	*Bifiobacterium bifidum* BGN4 and *Bifidobacterium longum* BORI	Capsule	Elderly men and women in Korea (aged ≥ 65)	53	1 × 10^9^ CFU/day	3 months	• Improved frequency of gas passage and abdominal distention • Improved mental flexibility (i.e., attention and executive function) • Alleviated stress • Increased serum BDNF	Decreased: *Eubacterium* *Clostridiales* *Allisonella* *Prevotellaceae*	Kim *et al.* (2021)^[217]^
	*Bifidobacterium breve* MCC1274	Powder	Elderly men and women with MCI in Japan (aged 65-89)	115	2 × 10^10^ CFU/day	24 weeks	• Improved cognitive orientation • Suppressed brain atrophy	No significant differences	Asaoka *et al.* (2022)^[218]^
	18-strain blend of *Lactobacillus*, *Lactococcus* and *Bifidobacterium* genera	Capsule	Elderly men and women with MCI in China (aged ≥ 60)	42	4 × 10^10^ CFU/day	3 months	• Enhanced cognitive function (i.e., attention, calculation, recall, executive function, visuospatial) • Improved sleep quality • Improved GI symptoms (e.g., abdominal pain, acid reflux, constipation)	Increased: *Bacteroidetes* *Ruminococcus* *Prevotellaceae* *Lachnospiraceae*	Fei *et al.*(2023)^[219]^
	*Bifidobacterium longum* BB68S	Powder	Healthy elderly men and women in China (aged 60-75)	50	5 × 10^10^ CFU/day	2 months	• Improved cognitive function (e.g., list learning, story recall, figure recall)	Increased: *Firmicutes* *Actinobacteria* *Bifidobacterium* *Lachnospira* Decreased: *Proteobacteria* *Eubacterium*	Shi *et al.* (2022)^[220]^

BMI: Body mass index; CFU: colony forming units; N/A: not applicable; MCI: mild cognitive impairment; BDNF: brain-derived neurotropic factor; GI: gastrointestinal.

The therapeutic evidence of probiotics for obesity and aging is undeniable, with an evident trend of gut modulation, regulated gut function, and improved systemic health (i.e., decreased inflammation and improved metabolism) following probiotic supplementation. However, it is important to highlight that the bioactivity and microbial modulatory properties of probiotics are often strain-specific. Thus, probiotics of interest must be studied for their specific influence on gut composition prior to their translation as therapeutics. Studies that evaluate the effect of probiotics on obesity and aging simultaneously, such as within sarcopenic obese models, are lacking. Further investigation is therefore required to elucidate the anti-obesity and pro-longevity potential of known and novel strains within the *Lactobacillus* and *Bifidobacterium* genera and beyond.

### Synbiotics

Prebiotics may also be combined with probiotics, in the form of a synbiotic, to exert a synergistic effect. Synbiotics are defined as “a mixture comprising live microorganisms and substrate(s) selectively utilized by host microorganisms that confers a health benefit on the host”^[222]^. Recent literature has investigated specific synbiotic combinations for obesity and aging. Notably, a novel synbiotic formulation comprised of probiotics *B. longum* spp. *infantis* NCIMB 702255, *L. fermentum* NCIMB 5221, and *L. plantarum* NCIMB 8826, and Triphala, a polyphenolic-rich Ayurvedic herb, was investigated by Westfall *et al.* for therapeutic effects on obesity and systemic metabolic markers^[187]^. Although the individual probiotics demonstrated beneficial effects, as discussed earlier, treatment with the synbiotic formulation resulted in the greatest impact. Specifically, in diet-induced obese *Drosophila*, beneficial variations in the gut microbiota (e.g., decreased *F/B* ratio), improved metabolic markers (e.g., lipogenesis, insulin resistance), and weight loss were most significant in flies treated with the probiotic or synbiotic formulations. Westfall *et al.* also studied the therapeutic potential of this synbiotic formulation in aging *Drosophila* and found it to have more robust combinatorial effects compared to its individual constituents, extending lifespan by a remarkable 60% and reducing metabolic parameters (e.g., total weight, total glucose, total triglycerides)^[223]^. Age-dependent elevations in inflammation and oxidative stress were also attenuated by the synbiotic formulation. These findings suggest that longevity and age-associated metabolic impairment and inflammation may be improved simultaneously with probiotics and prebiotics, or their combination, due to a synergistic effect. Another synbiotic formulation containing *L. acidophilus*, *B. lactis*, *B. longum*, *B. bifidum* and a prebiotic GOS mixture beneficially modulated the gut microbiota (e.g., increased abundance of *Lactobacillus* and *Bifidobacterium* and microbial diversity) in humans on a low-carbohydrate, high-protein, reduced energy diet, although body composition was not altered^[224]^. Along with prebiotics, probiotics may be combined with other nutraceuticals for health benefits. A recent clinical trial investigated the efficacy of supplementation with *Bacillus coagulans* combined with other nutraceuticals (i.e., antioxidant formulation, fish oil, cinnamon, and fiber supplement) in healthy overweight and obese subjects with cardiometabolic risk factors on a strict Mediterranean diet^[225]^. Findings revealed a significant improvement in longevity and cardiometabolic risk factors in treated subjects, including reduced serum lipids, inflammation (e.g., high-sensitivity CRP), blood pressure, insulin resistance, and a remarkable 50% reduction in diagnostic criteria for cardiometabolic syndrome and 38% decrease in Framingham 10-year cardiovascular risk. Supplementation of probiotics with fish oil and Vitamin D has also been investigated in overweight or obese individuals, resulting in decreased systemic inflammation after an eight-week treatment period^[226]^. A similar treatment was evaluated in a recent mother-infant clinical trial conducted in Finland^[227]^. Briefly, overweight and early pregnant women (< 18 gestational weeks; BMI ≥ 25 kg/m^2^) were treated with probiotics (*Lacticaseibacillus rhamnosus* HN001 and *Bifidobacterium animalis* ssp*. Lactis* 420; 10^10^ CFU/day), with or without the addition of fish oil capsules as a synbiotic, until six months post-partum. The effects of the probiotic and synbiotic treatments were investigated for their influence on the tendency to be overweight in the participants’ 24-month-old children. Findings reveal that the administered probiotic or synbiotic reduced the risk of overweight in their 24-month-old children, while no effect on body fat percentage was found. This study emphasizes the influence of gestational obesity and dietary intake on infant health, as well as the safety of probiotics and synbiotics.

Clinical evidence shows that specific synbiotic formulations promote the growth of beneficial gut bacteria and improve systemic health in obese or aged individuals^[224,228]^. For example, decreased systemic inflammation and positive effects on body weight were observed following treatment with a synbiotic (i.e., *B. lactis* and fructo-oligosaccharides) in obese women^[229]^. Synbiotic treatment may also improve overall GI symptoms, as evident in children with IBS, where synbiotic treatment was found to be superior to the administration of prebiotics alone^[230]^. However, as with the other biotic therapies, clinical studies are significantly lacking. Nevertheless, the potential of prebiotics and synbiotics for obesity and aging is promising and would benefit from further exploration.

### Postbiotics

Non-viable microorganisms and their byproducts may play a role in the health benefits of probiotics^[231]^. Such non-living organisms related to or derived from living organisms have emerged as a new concept, known as postbiotics. Recently, the International Scientific Association for Probiotics and Prebiotics (ISAPP) defined postbiotics as a “preparation of inanimate microorganisms and/or their components that confer health benefits on the host”. The benefits of postbiotics occur via a variety of mechanisms, both directly and indirectly. One notable form of postbiotics is SCFAs, which enable anti-inflammatory and antioxidative effects, as discussed previously. The production of SCFAs by microbes is desired for many indications, including obesity and healthy aging. In particular, butyrate and propionate are able to stimulate gut hormones and reduce food intake^[193]^. Supplementation of SCFAs has been shown to promote host energy expenditure, which is associated with an upregulation of thermogenic genes, and prevent body weight gain in HFD-fed mice^[164,232]^. A recent study also revealed that supplementation with SCFAs (i.e., acetate, butyrate and propionate) for two weeks in aged mice reduces inflammaging, oxidative stress, and metabolic alteration and improves LPS-induced acute lung injury through the gut-lung axis^[233]^. These findings support the crosstalk between the gut and distal organs as a therapeutic target. Several probiotic strains produce SCFAs and demonstrate beneficial effects on gut barrier function, including the upregulation of TJ proteins^[234]^. The benefits of *L. fermentum* NCIMB 5221 mentioned earlier may be partly due to its potent SCFA-producing abilities^[235]^. Additionally, ferulic acid (FA) is an antioxidant known to neutralize free radicals, such as ROS. Certain bacterial cells produce FA through the enzyme ferulic acid esterase (FAE), which may be particularly beneficial for obese-related dysbiosis^[236]^. FA exhibits anti-obesogenic effects, including reducing body weight and visceral fat accumulation, regulating appetite hormones, and reducing HFD-induced inflammation^[237]^. FA is also proposed to promote healthy longevity through Sirt1 activation, subsequent autophagy, and induction of antioxidant enzymes, among other aging hallmarks, such as inflammation and cellular senescence^[238]^. Additionally, bile salts play a critical role in metabolic homeostasis through the regulation of cholesterol levels and fat metabolism^[239]^. Disordered BAs are also associated with aging. Specific intestinal bacteria, such as the aforementioned *L. plantarum* WCFS1, produce BSH, which deconjugates bile salts and enables the release of free BAs and amino acids, while lowering cholesterol^[240]^. Other metabolic products of the gut, such as vanillic acid and linoleic acid, have also been proposed as promising postbiotics for obesity due to reports of inducing thermogenesis and increasing energy expenditure, respectively^[193]^. Additionally, many lactic acid bacteria produce antimicrobial peptides (AMPs; e.g., bacteriocins) that are able to avert pathogenic bacteria associated with obesity and aging^[241,242]^. Moreover, bacterial-derived exopolysaccharides (EPS) have gained interest as prebiotics and demonstrated anti-obesogenic effects^[243]^, likely via intestinal microbiota modulation^[244]^. EPS from *L. acidophilus, L. gasseri, L. plantarum,* and *L. rhamnosus* show significant health-promoting benefits, including antioxidant properties in an aging mouse model and antitumor activity^[245]^. Overall, there are numerous postbiotics that can be studied and leveraged as novel therapeutics based on their action. Currently, available evidence suggests that many postbiotics have great promise as therapeutics for obesity and aging, warranting additional exploration.

### Limitations of biotic therapeutics and their investigation

Although hopeful, the use of the microbiome, probiotics, synbiotics, and other biotic forms as next-generation therapeutics faces many obstacles. Our diverse ecosystem is home to a near-infinite number of microbes and natural polyphenols with potential prebiotic activity, although not yet discovered. This provides a great opportunity but also necessitates procedures for researching such materials. For pre- and probiotics already identified and studied, guidelines on therapeutic dosages are lacking. Similarly, determining how best to combine different probiotics and create synbiotic formulations for maximal synergy remains unknown^[193]^. The lack of consensus may be partly attributed to the various research methods used to study biotics, as well as the wide range of agents studied. Biotic-focused studies can be improved for future studies. For example, quercetin alone may be investigated as an aging therapy without the addition of Dasatinib. Importantly, pre-clinical and clinical trials of biotics should standardize the analysis of the gut microbiota as a study outcome. This can also be extended to observational studies on obese and aged populations to help reach a consensus on gut microbiota alterations associated with these diseases. Methods for the collection and analysis of microbiota samples must also be standardized. Many animal studies isolate microbiota from intestinal tissue samples, while clinical trials rely on fecal samples. These differences may contribute to the high variability seen in the published data on gut microbial composition. Moreover, many of the recent clinical trials for obesity and aging were conducted in Asia, which may not be applicable to the Western populations, given the different gut microbiota among these regions. In addition, variability in diet and exercise can impact results from clinical trials that investigate biotic therapies. The diagnostic criteria for overweight or obese also vary between studies, as does the criteria for an elderly age. Standardization of such factors would provide more widely applicable data. Moreover, to our knowledge, there are no clinical trials on the sarcopenic obese population. The shortage of clinical studies is a leading limitation for the translation of biotic therapies. The majority of the reviewed clinical trials had a treatment duration of three months, which does not reflect the safety of biotic therapies. Long-term investigation of biotic supplementation is therefore warranted. In addition, the investigation of biotics with therapeutic effects for obesity should be considered as a treatment for aging studies, and vice versa. Such studies would advance understanding of the link between obesity and aging and may discover biotics that can be used for both diseases. Overall, additional short- and long-term human trials investigating different biotics for general health and disease states, such as obesity, unhealthy aging, and sarcopenic obesity, are required to demonstrate the clinical efficacy of the various biotic forms.

## CONCLUSION

The abundance of microorganisms that inhabit the human body and their critical roles in human health and disease present unlimited potential for microbiome-engineered therapeutics. Obesity and aging are complex diseases that are together rising in prevalence, increasing the global risk of debilitating and chronic illnesses. Many hallmarks are shared between obesity and aging, one of which is gut microbial dysbiosis. The gut microbiota may be targeted to re-establish a healthy flora in obese or aged individuals, thereby improving the disease phenotypes, underlying metabolic dysfunction and systemic comorbidities. Importantly, the unique relationship between obesity and aging may be targeted with the use of biotic therapies through the well-established GBA. The microbiome and various forms of biotics - namely prebiotics, probiotics, synbiotics, and postbiotics - offer distinct benefits to the host, which can be exploited to develop novel therapeutics for obesity, aging, and related diseases. Several areas of research can be further explored to establish and engineer biotics, including their mechanisms of action, strain-specific therapeutic effects, and their combinations as synergistic therapies. Future studies may advance the literature on the use of postbiotics in obesity and aging by studying the effects of probiotic products on other shared hallmarks, such as adipose tissue dysfunction. Regulation of appetite hormones by biotics may also be studied. In addition, investigating the mechanisms of the GBA in sarcopenic obesity may help identify specific pathways and markers that link obesity and aging through the GBA. Specifically, vagal activity should be evaluated in obese, aging, and comorbid populations. Advancing knowledge on prominent diseases like obesity and aging through research on biotics would pave the way for groundbreaking medicine. The era of microbiome-tailored therapies has only just begun and biotic therapeutics remain at the forefront.

## DECLARATIONS

### Copyright

© The Authors 2024.
